# Highly Pathogenic Avian Influenza Virus among Wild Birds in Mongolia

**DOI:** 10.1371/journal.pone.0044097

**Published:** 2012-09-11

**Authors:** Martin Gilbert, Losolmaa Jambal, William B. Karesh, Amanda Fine, Enkhtuvshin Shiilegdamba, Purevtseren Dulam, Ruuragchaa Sodnomdarjaa, Khuukhenbaatar Ganzorig, Damdinjav Batchuluun, Natsagdorj Tseveenmyadag, Purevsuren Bolortuya, Carol J. Cardona, Connie Y. H. Leung, J. S. Malik Peiris, Erica Spackman, David E. Swayne, Damien O. Joly

**Affiliations:** 1 Wildlife Conservation Society, Bronx, New York, United States of America; 2 EcoHealth Alliance, New York, New York, United States of America; 3 State Central Veterinary Laboratory, Transboundary Animal Disease Laboratory, Avian Influenza Section, Ulaanbaatar, Mongolia; 4 Mongolian Academy of Sciences, Institute of Biology, Ulaanbaatar, Mongolia; 5 Avian and Human Influenza Project, World Bank, National Emergency Management Agency, Ulaanbaatar, Mongolia; 6 University of Minnesota, Department of Veterinary and Biomedical Science, Saint Paul, Minnesota, United States of America; 7 The University of Hong Kong, School of Public Health, Department of Microbiology, Hong Kong, People’s Republic of China; 8 United States Department of Agriculture Agricultural Research Service, Southeast Poultry Research Laboratory, Athens, Georgia, United States of America; Duke-NUS Graduate Medical School, Singapore

## Abstract

Mongolia combines a near absence of domestic poultry, with an abundance of migratory waterbirds, to create an ideal location to study the epidemiology of highly pathogenic avian influenza virus (HPAIV) in a purely wild bird system. Here we present the findings of active and passive surveillance for HPAIV subtype H5N1 in Mongolia from 2005–2011, together with the results of five outbreak investigations. In total eight HPAIV outbreaks were confirmed in Mongolia during this period. Of these, one was detected during active surveillance employed by this project, three by active surveillance performed by Mongolian government agencies, and four through passive surveillance. A further three outbreaks were recorded in the neighbouring Tyva Republic of Russia on a lake that bisects the international border. No HPAIV was isolated (cultured) from 7,855 environmental fecal samples (primarily from ducks), or from 2,765 live, clinically healthy birds captured during active surveillance (primarily shelducks, geese and swans), while four HPAIVs were isolated from 141 clinically ill or dead birds located through active surveillance. Two low pathogenic avian influenza viruses (LPAIV) were cultured from ill or dead birds during active surveillance, while environmental feces and live healthy birds yielded 56 and 1 LPAIV respectively. All Mongolian outbreaks occurred in 2005 and 2006 (clade 2.2), or 2009 and 2010 (clade 2.3.2.1); all years in which spring HPAIV outbreaks were reported in Tibet and/or Qinghai provinces in China. The occurrence of outbreaks in areas deficient in domestic poultry is strong evidence that wild birds can carry HPAIV over at least moderate distances. However, failure to detect further outbreaks of clade 2.2 after June 2006, and clade 2.3.2.1 after June 2010 suggests that wild birds migrating to and from Mongolia may not be competent as indefinite reservoirs of HPAIV, or that HPAIV did not reach susceptible populations during our study.

## Introduction

Since its emergence in 1997 and subsequent re-emergence in 2003, highly pathogenic avian influenza virus (HPAIV) subtype H5N1 has caused the deaths of at least 357 people in 15 countries [1] and been responsible for losses of many millions of domestic poultry, negatively impacting economic growth and food security in affected countries. Prior to 2005, outbreaks in wild birds were sporadic, associated with high mortality, and thought to relate to spillover from infected domestic poultry [2]–[6]. This situation changed in April 2005, with an outbreak among wild migratory waterbirds at Qinghai Lake in northern China, when over 6,000 wild birds died over a period of two months [7]–[9].

Following events at Qinghai, there was a marked increase in outbreaks involving wild birds elsewhere in Asia, Europe and Africa. However, the extent to which wild birds contributed to the spatial expansion of HPAIV outbreaks, particularly in relation to that of the legal and illegal movement of wild and domestic fowl is difficult to resolve [10], [11]. Many outbreaks involving wild birds occurred in close proximity to cases among domestic poultry [12], making it difficult to attribute the source of infection to a wild or domestic host. In such environments, it can be difficult to isolate the contribution that wild birds play in cycles of HPAIV transmission [13].

In response to the Qinghai outbreak, a wild bird surveillance system was implemented in Mongolia in 2005 to elucidate the role of wild birds in the transmission of HPAIV H5N1. Bird species show variable susceptibility to infection with HPAIV H5N1, with experimental exposures resulting in high mortality rates among swans, geese and gulls [14], [15], and variable levels of mortality or subclinical infections in ducks [15]–[17] Active surveillance approaches involved searching for the presence of HPAIV through screening diagnostic specimens from healthy, ill and dead birds and from the environment. In contrast, passive surveillance relied on reports of sick and dead birds received from the general public, who were requested to report waterbird mortality to provincial veterinary authorities.

Several features make Mongolia an ideal location for understanding the epidemiology of HPAIV in wild birds. Mongolia supports large populations of migratory waterfowl and shorebirds [18], including species breeding across Mongolia’s extensive wetlands during the boreal summer, then departing to spend the winter in milder climates in Australasia, the Indian Subcontinent, Africa, Southeast Asia, China, Korea, and Japan [19]–[23]. HPAIV H5N1 is either endemic or has occurred in domestic and/or wild bird populations in all these areas except for Australasia. Other species visit Mongolia for short periods to feed while migrating to and from more northerly breeding areas. Mongolia also represents an important site for molting Anseriformes (ducks, geese and swans) that congregate during the post-breeding period when early frosts force them to vacate their Siberian breeding sites.

Mongolia presents an opportunity to study virus in wild populations in isolation from domestic poultry. It has a small domestic poultry industry with a population of fewer than 100,000 birds in 2005 [24], an overall density of <1 km^−2^ [25], with most birds raised for egg production in moderately biosecure facilities located in urban centers. In contrast, China produces approximately 5,900,000,000 domestic birds annually [26]. To date, Mongolia’s modest poultry population has remained free from HPAIV outbreaks. With little backyard production, the potential for wild birds being exposed to domestic sources of virus is negligible, allowing conclusions to be drawn on the status and transmission of HPAIV in wild bird populations without the complication of local spillover from domestic hosts.

Herein we report the seven-year findings of this surveillance program, including the success of various approaches of active and passive surveillance in detecting HPAIV outbreaks, together with the results of investigations once they were detected. Isolates of low pathogenic avian influenza viruses (LPAIV) in Mongolia have been reported elsewhere [27], and so will only be summarized briefly here. The implications of the project findings are discussed both with respect to future surveillance efforts, as well as to understanding the epidemiology of HPAIV in wild bird populations.

## Methods

### Study Area

Lying between the latitudes of 42°N and 51°N, Mongolia is a vast, land-locked country of approximately 1.5 M km^2^, that extends east-west across 2,500 km of Central Asia between Russia and China. Although a country of climatic and geographic extremes, much of the land area consists of open steppe, transitioning to taiga forest in the north and the Gobi Desert in the south and west. Wetlands and lakes of variable size fleck the landscape, with approximately 3,000 rivers stretching over 67,000 km in the north [28]. Mongolia is populated by just over three million people, over half of which depend directly or indirectly on the traditional nomadic system of livestock production, raising goats, sheep, cattle, yaks and horses [29].

### Active Surveillance

Active surveillance was directed at the detection of HPAIV outbreaks (defined for the purposes of the study as presence of one or more clinically ill or dead birds from which HPAIV could be isolated), and the presence of HPAIV in clinically unaffected birds. Detection of outbreaks relied on the collection of samples from dead or ill birds located along shoreline transects; while collection of samples from live birds showing no clinical signs of illness and environmental fecal deposits were used to detect the subclinical presence of virus. The approaches used each year are summarized in Table 1. Surveys were conducted in four main regions, the ‘east’ (referring to Ulaanbaatar and the aimags (provinces) of Dornod, Khentii and Sukhbaatar), the ‘west’ (Uuvs and Khovd), the ‘south-central’ (Bayankhongor, Zavkhan and Gov-Altai), and the ‘north-central’ (Khovsgol, Bulgan and Arkhangai).

**Table 1:** Summary of active HPAIV surveillance approaches employed in Mongolia, from 2005–2011.

Activity	2005	2006	2007	2008	2009	2010	2011
Live bird sampling	Limited	Yes	Yes	Yes	Yes	Yes	No
Faecal sampling?	Yes	Yes	No	No	Yes	Yes	Yes
Mortality transects?	Yes	Yes	Yes	Yes	Yes	Yes	No
Number of survey sites	9	42	12	10	11	23	17
Geographic regions	NC, SC	E, W, NC, SC	NC	NC	W, NC	E, NC	E
Timing	Jul-Aug	Jul-Oct	Apr-Oct	May-Sep	Jun-Sep	May-Sep	May- Oct

Geographic regions are East (E), West (W), North-central (NC), and South-central (SC).

### Live Bird Sampling

Live, apparently healthy birds were captured using a number of methods; none were killed for the purposes of this study. Live bird sampling focused on waterbirds particularly Anseriformes (including *Anas* ducks, shelducks, geese and swans), and Ciconiiformes (including gulls, cormorants and shorebirds). Molting Anseriformes were captured in groups, by driving birds into temporary holding pens, or individually at night with use of spotlights and nets or swan hooks. Shorebirds were caught at night using mist nets, and hand nets were used to catch cormorants and gulls. Tracheal and cloacal swabs were collected from each bird and stored individually in cryovials containing viral transport media (VTM). Oropharangeal swabs were collected instead of tracheal swabs from smaller bird species. Samples were held at 4°C up to four hours and then immersed in liquid nitrogen. Cold chain was maintained throughout delivery of samples to the laboratory. In 2006, 2007 and 2008, a duplicate set of respiratory and cloacal samples were collected and stored in cryovials containing 10% guanidine isothiocyanate solution (2007), or a solution of 1% Environ One-Stroke (2006 and 2008), for molecular analysis.

### Dead/sick Bird Sampling

Shoreline transects were delineated at each site and systematically searched for sick and dead birds. Where possible they completely circumnavigated the water body, or at least exceeded 5 km on larger lakes. Birds were identified when possible to species, sex, age and general approximate time since death. Obvious signs of predation or scavenging were recorded and a photo record made of each bird. Where species could not be determined due to carcass condition, identity was recorded to the highest taxonomic level possible or simply listed as “unknown”. Swab samples were collected in the same manner described for live birds. Where possible, tissues from brain, lung, spleen and pancreas were also snap frozen in VTM for virus isolation.

### Fecal Sampling

Fresh fecal samples were collected where waterbirds were observed congregating or roosting. Samples were collected and preserved in the same manner as those collected from live birds. In 2005 and 2006 sampling focused on single-species flocks to enable the identification of the species being sampled. This led to a bias toward certain species that were less likely to form mixed-species groups, particularly shelducks, geese, swans, gulls and cormorants. From 2009 onward, our collection strategy changed to include species that habitually congregate in mixed-species groups (particularly focusing on ducks in the genera *Anas* and *Aythya*, which are prominent carriers of LPAIVs), sacrificing specific identity of the birds being sampled.

### Passive Surveillance

Reports of sick and dead birds from herders, local veterinary, or environmental officials were relayed via the district and province veterinary offices to the State Central Veterinary Laboratory (SCVL) in Ulaanbaatar, with samples or whole birds collected by national or provincial investigation teams submitted for laboratory testing at SCVL. Samples collected from suspect cases were forwarded to the OIE Reference Laboratory at Hokkaido University for confirmation.

### Outbreak Investigation

Reports of suspected outbreaks were relayed to the study team to enable follow-up outbreak investigations to take place. Standardized counts were performed at each outbreak site using recognized guidelines [30]. Point counts were supplemented with active searching to provide a more comprehensive list of species present at each site. Where feasible, attempts were made to capture and sample live birds at outbreak sites, and to collect fecal samples from areas where birds were congregating.

### Diagnostics

All samples were submitted for virus isolation, except in 2006, and 2007 when inactivated duplicate samples were screened using real-time RT-PCR (rRT-PCR) [31]. Corresponding samples in VTM to those positive by rRT-PCR were then submitted for virus isolation. In 2008 all samples were screened using virus isolation of VTM samples and rRT-PCR of inactivated duplicates. Virus isolation was accomplished in 10-day old specific-pathogen-free embryonated chicken eggs using described procedures [32]. Hemagglutinating agents from virus isolation attempts were confirmed as type A influenza by antigen capture and rRT-PCR [33]. Viruses were pathotyped using chicken pathogenicity tests and sequencing of the H5 hemagglutinin proteolytic cleavage site [32], [34].

Processing of samples using RT-PCR took place in several laboratories, therefore the procedures were not identical. In 2006 total RNA was extracted with a procedure optimized for cloacal swab samples. The RNA was screened for AIV by rRT-PCR using a standard protocol [33]. The rRT-PCR test was run with an internal positive control [35] to ensure that inhibitors were not causing false negative results. All rRT-PCR positive samples were processed for virus isolation in embryonated chicken eggs and were screened for H5 subtype virus with subtype specific primers and probes [36], [37]. Samples from 2007 and 2008 were processed at the University of California, Davis as follows: RNA was extracted from swab samples using the MagMAX- 96 Viral Isolation Kit (Ambion Inc. Austin, TX) in accordance with the manufacturer’s instructions. An rRT-PCR which targets the M gene was conducted as described by Runstadler, et al. [38], [39] which was the standard test in this laboratory and run on an AB 7500 Real-Time PCR System (Applied Biosystems, Foster City, CA).

Amnio-allantoic fluid from eggs inoculated for virus isolation, testing positive for hemagglutination, an indicator virus presence, were processed for RNA and run on the AIV type A rRT-PCR to confirm virus presence. Regardless of the initial rRT-PCR result the subtypes of all isolates were confirmed by sequencing [27]. We assumed an individual was negative for HPAIV H5N1 if all tests conducted on samples from that individual were negative.

### Ethics Statement

Bird capture and handling permits covering all four regions of Mongolia (east, west, north-central and south-central) were issued by the Mongolian Ministry of Nature, the Environment and Tourism following approval of handling and sampling protocols by the Institute of Biology at the Mongolian Academy of Sciences, with additional approval from the University of Minnesota, Institutional Animal Care and Use Committee (Protocol 1006A84613).

## Results

In total, eight outbreaks of HPAIV were recorded in Mongolia during the study period (Table S5), with three more occurring within Russian territory along the shores of Uuvs Nuur, a lake that bisects the international boundary (Table S7). These eleven outbreaks occurred at just five sites (with three outbreaks each recorded at Erhel Nuur and Uuvs Nuur). An additional report of three dead whooper swans found at Zegst Nuur in Sukhbaatar province on 5 April 2011 (Ref OIE: 10500) was not considered to constitute a confirmed outbreak, as the diagnosis was based on RT-PCR and rRT-PCR for H5 only, and no virus could be isolated.

### Active Surveillance

**Figure 1:**
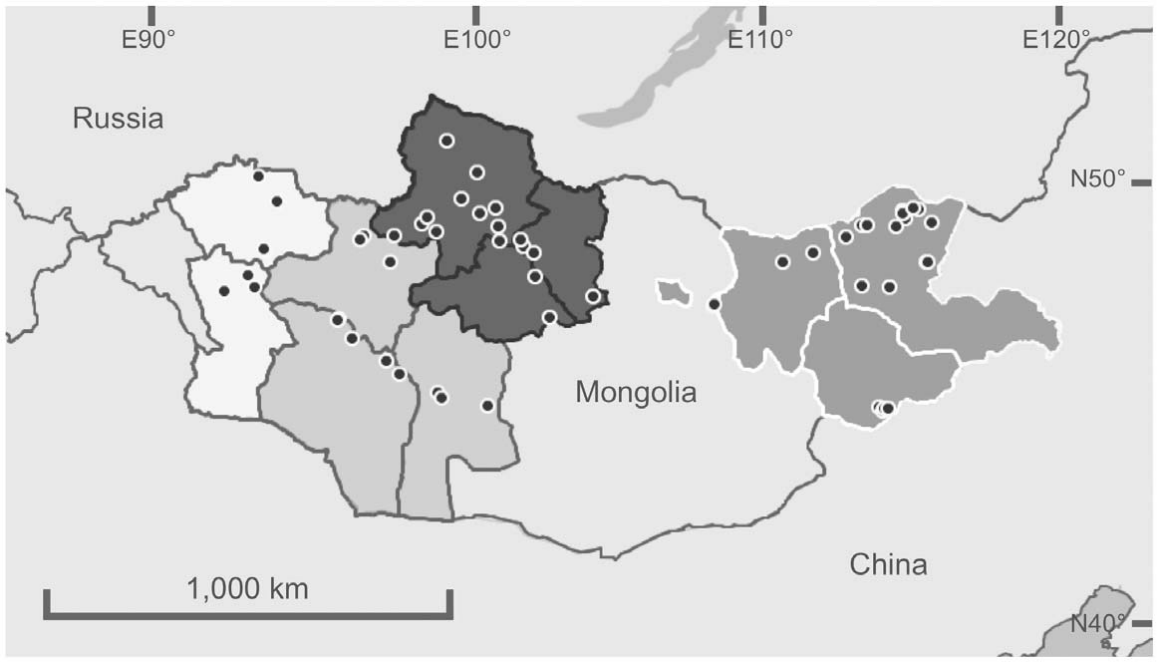
Map of study sites. Boundaries of study regions illustrated (white = West; light grey, white border = East; dark grey = North-central; light grey, dark border = South-central).

Active surveillance was conducted in all seven years at 61 locations (Figure 1, Table 1). Overall, 10,761 individual birds were sampled using active surveillance (Table 2, and Tables S1 and S3), but only one outbreak of HPAIV H5N1 was identified using the methods employed by this study, at Erhel Nuur in the north-central region on 28 July 2009, when isolates of HPAIV H5N1 were recovered from three dead juvenile Mongolian gulls *Larus mongolicus*. Although the outbreak at Erhel Nuur in 2009 was detected during active surveillance, further details are provided in the section reporting the results of outbreak investigations for comparative purposes. A further three outbreaks were detected by active surveillance undertaken by Mongolian government agencies at Khunt Nuur (May 2006), Erhel Nuur (May 2006) and Doroo Tsagaan Nuur (July 2009).

**Table 2:** Summary of the number of individual birds sampled during active surveillance in Mongolia 2005–2011.

Surveillance type	Order	2005	2006	2007	2008	2009	2010	2011	Grand Total
Live bird	ANSERIFORMES	0	8	315	794	731^a^	48	0	1,896
	CICONIIFORMES	0	9	148	475	160^a^	0	0	792
	PICIFORMES	0	0	0	0	0	2	0	2
	PASSERIFORMES	0	8	0	0	0	67	0	75
Faecal	ANSERIFORMES	413	1,265	0	0	720	1,521	2,400	6,319
	GRUIFORMES		15	0	0	0	0	0	15
	CICONIIFORMES	35	1,345	0	0	140	0	0	1,520
	PASSERIFORMES	0	1	0	0	0	0	0	1
Sick/dead	ANSERIFORMES	1	10	4	1	3^b^ [1]	0	0	19
	CICONIIFORMES	0	15	40	52	8^b^ (3)	1	0	116
	FALCONIFORME	0	1	0	0	0	0	0	1
	UPUPIFORMES	0	0	1	0	0	0	0	1
	PASSERIFORMES	0	1	0	0	3	0	0	4
	Total	449	2,678	508	1,322	1,765	1,639	2,400	10,761

In 2008 samples were submitted for culture in embryonated eggs, and RT-PCR, the results obtained for culture are represented here.

HPAIV H5N1 viruses identified by virus isolation (VI) are indicated in parentheses, and those identified by real time reverse transcription polymerase chain reaction (PCR) are indicated in square brackets.

a Figures include 197 live, clinically healthy Anseriformes, and 83 Ciconiiformes sampled on Erhel Nuur between 28 July and 1 August 2009, a period that coincided with an outbreak of HPAIV H5N1 that was subsequently confirmed through laboratory analysis.

b Figures include one sick ruddy shelduck, and three dead juvenile Mongolian gulls at Erhel Nuur in July-August 2009 that subsequently tested positive for HPAIV H5 by rRT-PCR, and HPAIV H5N1 by virus isolation respectively.

A total of 56 LPAIVs were isolated from environmental fecal samples (n = 7,855), one from live clinically healthy birds (n = 2,765) and two from dead or ill birds (n = 141) sampled during active surveillance. In 2006 6.9% of samples tested positive for the matrix gene (n = 2,678), compared to 9.4% in 2007 (n = 508) and 9.4% (n = 1,377) (Table S3). Of 363 samples testing positive for matrix sequences only two tested positive for H5 (a fecal sample from a red crested pochard, *Rhodonessa rufina*, and a cloacal sample from a red-necked stint *Calidris ruficollis*), but no virus was isolated from the duplicates of these samples. In 2008 when all samples were tested by both RT-PCR and virus isolation, only one LPAIV was isolated, from a sample testing negative by RT-PCR for the matrix gene (from a live ruddy shelduck *Tadorna ferruginea*).

### Passive Surveillance

Of the eight Mongolian outbreaks, four were detected through passive surveillance at Khunt Nuur (July 2005), Erhel Nuur (July 2005), Doitiin Tsagaan Nuur (May 2009) and Ganga Nuur (May 2010) (Table S5).

### Outbreak Investigation

Five outbreak investigations were completed during this study, including Erhel Nuur (June 2005), Uuvs Nuur (June 2009), Doroo Tsagaan Nuur (August 2009), and Ganga Nuur Nature Reserve (May 2010). Although the outbreak at Erhel Nuur in 2009 was identified during active surveillance, activities performed were consistent with those at outbreak investigations and are reported here.

Population numbers, densities and species identification of live birds were obtained during each of the five outbreak investigations (Table S4). Due to the size of Uuvs Nuur, we were unable to survey birds across the entire surface area of the lake. We estimated the area surveyed at Uuvs Nuur, by assuming that all birds could be reliably identified and counted up to 2 km from the shoreline (estimated on-site using maps), and multiplying this against the length of shoreline surveyed. Counts made at all other sites represent the entire surface area of the lakes. Ganga Nuur Nature Reserve data combines surveys of its main water bodies (Ganga Nuur, Kholbo Nuur, Erdene Nuur and Huuvur Nuur).

Fifteen sick or dead birds suitable for sample collection were identified during outbreak investigations, with a further four located at Erhel Nuur in July 2009 (Table 3 and Table S2). HPAIV H5N1 virus was isolated from eight of these birds, with an additional three testing positive for HPAIV subtype H5 virus by rRT-PCR. Among birds testing positive by rRT-PCR was a dead great crested grebe *Podiceps cristatus* at Uuvs Nuur in June 2009. This bird, found 19.2 km from the border with the Russian Federation, remains the only case of HPAIV H5 on the Mongolian shores of Uuvs Nuur.

**Table 3:** Summary of the number of individual birds sampled during outbreak investigations in Mongolia 2005–2011.

Surveillance type	Order	2005	2006	2007	2008	2009	2010	2011	Grand Total
Live bird	ANSERIFORMES	1	0	0	0	91	0	0	92
	CICONIIFORMES	1	0	0	0	0	0	0	1
Faecal	ANSERIFORMES	412	0	0	0	141	151	0	704
	CICONIIFORMES	19	0	0	0	0	0	0	19
Sick/dead	ANSERIFORMES	4 (1)	0	0	0	4 (3)	1 (1)	0	9
	CICONIIFORMES	2	0	0	0	4 [1]	0	0	6
	Total	439	0	0	0	240	152	0	831

HPAIV H5N1 viruses identified by virus isolation (VI) are indicated in parentheses, and those identified by real time reverse transcription polymerase chain reaction (PCR) are indicated in square brackets.

Capture of live, clinically healthy birds was only possible during the outbreak investigations at Erhel in 2005 (2 birds) and Doroo Tsagaan Nuur in 2009 (91 birds) (Table 3 and Table S2). A further 279 live birds were captured during active surveillance at Erhel Nuur in July 2009 (Table 2 and Table S1) which was later identified as an outbreak. No HPAIV H5N1 virus was isolated from any of these birds.

A total of 723 fecal samples were collected during outbreak investigations, from which no isolates of HPAIV H5N1 virus were recovered.

## Discussion

Although confirmed outbreaks of HPAIV, subtype H5N1 in Mongolia occurred during four of the seven study years, our active surveillance was only successful in identifying one outbreak (Erhel Nuur, July 2009). In six of the seven years of surveillance sampling of live birds (either through capture or collection of fecal samples) concentrated on ruddy shelducks, bar-headed geese *Anser indicus* and whooper swans *Cygnus cygnus* as these species were over-represented among dead birds found at outbreaks (Tables S1, S2, S3 and S5). A total of 2,504 of these birds were sampled during active surveillance, and 503 during outbreak investigations, but no HPAIV was isolated. Duck species within the genera *Anas* and *Aythya* that may be more likely to harbor subclinical HPAIV infections [15], [17] were under-represented during captures, which led to the decision to increase collections of fecal samples from these species in 2009, 2010 and 2011. A total of 4,401 samples were collected from these species during active surveillance and 292 during outbreak investigations, but also failed to yield HPAIV isolates. Taken together these findings suggest that sampling of live birds (through capture and fecal sampling) are either insensitive at detecting outbreaks, or that the species targeted were inappropriate.

Although considerably fewer samples were collected from ill and dead birds, these yielded HPAIV isolates both during active surveillance (three juvenile Mongolian gulls at Erhel Nuur in July 2009) and outbreak investigations (one whooper swan at Erhel Nuur in 2005, two ruddy shelducks and a bar-headed goose at Doroo Tsagaan Nuur in 2009, and one tundra swan *Cygnus columbianus* at Ganga Nuur in 2010). Since at least half of the confirmed Mongolian outbreaks were identified by means of passive surveillance, and sick and dead bird sampling accounted for all HPAIV isolates, passive surveillance and investigation of mortality incidents may be the most cost-effective method of identifying outbreaks of HPAIV in wild birds in Mongolia and possibly other countries. However, with 59 LPAIVs isolated during active surveillance, the wider value of this approach to understanding influenza ecology should not be discounted.

Due to the variation in strategies of laboratory analysis used during the study, it is conceivable that our ability to detect HPAIV may have been lower in some years compared to others. In particular, the pre-screening of duplicate samples using RT-PCR in 2006 and 2007 may have resulted in missing viruses among samples that were negative by RT-PCR and therefore not submitted for virus culture. Indeed, in 2008 an LPAIV was isolated from a ruddy shelduck that tested negative using RT-PCR exemplifying this possibility. However, overall RT-PCR was found to be more sensitive than virus culture in detecting viruses, with 363/4,563 (7.96%) found positive by RT-PCR compared to 79/8,406 (0.94%) by virus isolation. This is not unexpected, as unlike virus isolation, RT-PCR will detect both viable and non-viable virus.

**Figure 2:**
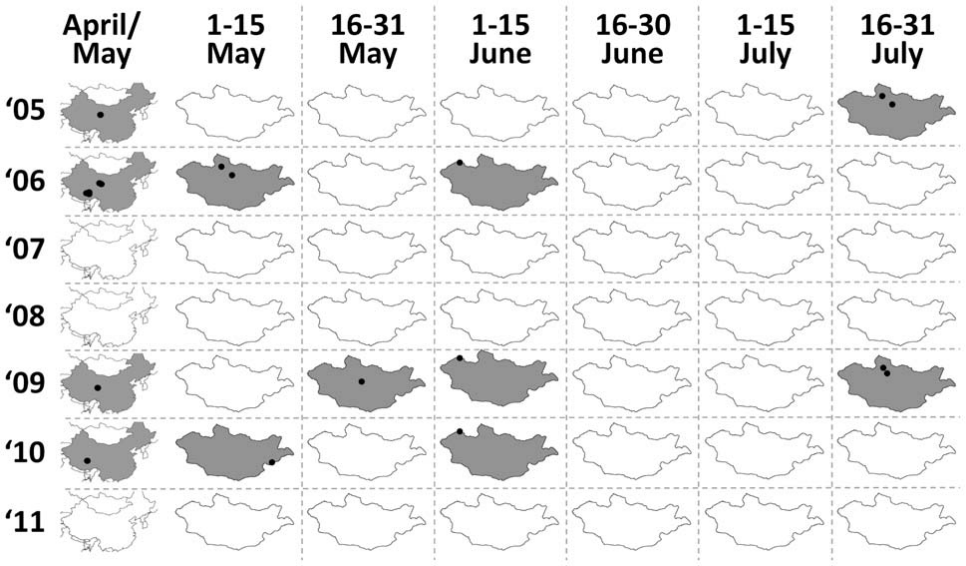
The spatiotemporal distribution of HPAIV outbreak onset from 2005–2011. All reported outbreaks are illustrated for the People’s Republic of China during April and May (left column), and Mongolia, in two week intervals from May – July. Cells corresponding to periods during which outbreaks were first reported are indicated in grey, and locations of outbreaks are indicated in black.

The low density of poultry in Mongolia, coupled with the lack of any recorded outbreaks among Mongolian poultry strongly supports the hypothesis that wild birds are responsible for carrying HPAIV H5N1 to Mongolia. Furthermore, Mongolian cases only occurred in years when wild bird outbreaks were also reported in either Qinghai or Tibet during April or May (Figure 2; Table S7). The species that predominated in the Qinghai and Tibet outbreaks were similar to those that died in Mongolia (with the exception of brown-headed gull *Chroicocephalus brunnicephalus* which is uncommon in Mongolia). All isolates obtained from Mongolia in 2005 and 2006 fell within the same subclade as those obtained from Qinghai and Tibet during the same period (clade 2.2), while those obtained in 2009 and 2010 also clustered with viruses obtained from the Qinghai and Tibetan outbreaks within these years (clade 2.3.2.1). This trajectory of outbreaks is consistent with wild birds migrating northwards along the Central Asian migratory flyway during the spring, a route that is followed by birds wintering in southwestern China and the Indian subcontinent. Notably, these clades have also been isolated among domestic poultry in India (clade 2.2), Nepal (clade 2.3.2.1) and Bangladesh (both clades) [40]–[42]. Further isolates of clade 2.3.2.1 viruses among wild birds in Hong Kong in the winter of 2007/8 [43], in Japan during spring 2008, and again in Japan and the Korean Peninsula during the winter 2010/11 [44] suggest that this clade has also been moving along the East Asian Australasian flyway, at least to the latitude of southern China. HPAIV appears to have persisted in wild birds in the Central Asian migratory flyway for a minimum of 15 months in the case of both clade 2.2 and clade 2.3.2.1 (Tables S5, S6, S7), although the possibility of multiple reintroductions from infected domestic poultry elsewhere in the flyway cannot be discounted. However, based on the lack of any isolates of clade 2.2 from wild birds in the Central Asian flyway after June 2006, or clade 2.3.2.1 after June 2010 we hypothesize that while wild bird populations can support HPAIV infections for a limited period, they have not represented a competent reservoir in the long term. Testing this hypothesis would require that surveillance effort be increased across the flyway, with an emphasis on the reporting and investigation of dead birds.

The timing and location of HPAIV outbreaks on Mongolian waterways have shown a generally similar temporal and spatial distribution (Figure 2), with all outbreaks starting between early May and the end of July, and restricted to six locations. While the narrow spatial and temporal distribution of Mongolian outbreaks could be related to biases in surveillance locations, we do not believe this is the case. Combined with passive surveillance, which exhibits less temporal bias, Mongolian authorities have also implemented an extensive active surveillance program since 2006, targeting 97 wetland areas across 12 provinces, taking place at least twice a year.

**Figure 3:**
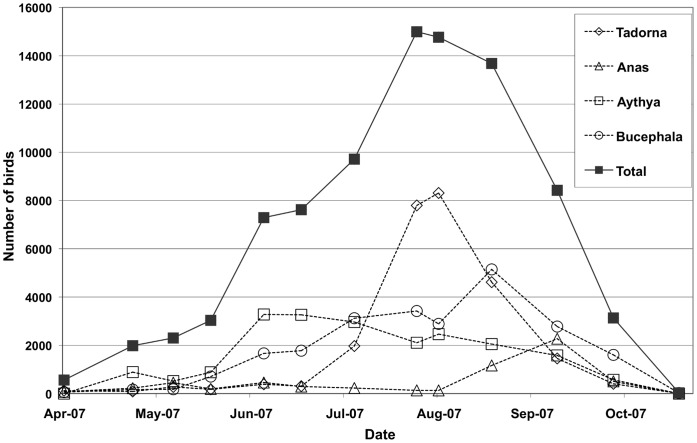
Monthly bird counts at Erhel Nuur from April to October 2007. Total number of birds present are indicated (solid squares), together with counts of the predominant genera *Tadorna* (open diamond), *Anas* (open triangle), *Aythya* (open square), and *Bucephala* (open circle).

Ecological factors are likely to play an important part in the seasonal distribution of outbreaks. Outbreaks in early May coincide with the arrival of spring migrants from southern wintering areas, including China and South Asia (mostly Anseriformes), and further south for many other species. Counts of birds obtained from April through October at Erhel Nuur in 2007 (Figure 3) illustrate how bird numbers vary during the period from spring thaw to autumn freeze. Although absolute numbers of birds are relatively small in early May, the continuation of ice coverage forces waterbirds into small areas of open water, which increases their density more than at other times of year. For instance, a total of 2,062 birds counted on Erhel Nuur on 5 May 2007 (a date equivalent to the outbreak the previous year) equates to a density of 2,222 birds km^−2^ based on a visual estimate that 95% of the lake surface was frozen. This is higher than the bird densities recorded at any other time at this site. Furthermore, in early May melting ice reduces the salinity of surface water to near zero (even on lakes that are markedly brackish for the remainder of the summer) (pers. obs.). However this effect is temporary and once all ice has melted (by mid to late May) the salinity rapidly increases and remains at a near constant until the autumn freeze. This dilution effect, coupled with low water temperatures may enhance the environmental survival of virus at this time of year [45].

The temporal delay in spring outbreaks in Uuvs Nuur (which have all occurred in early June) from those in north-central Mongolia are hard to explain. By early June spring migration is essentially over, and birds are focused on nest building and raising young. It may be that differences in the species assemblage (Table S4), or peculiarities in breeding or feeding ecology at Uuvs Nuur account for this difference (e.g. Uuvs Nuur supports large colonies of several species such as great cormorant *Phalacrocorax carbo*, Pallas’s gulls *Larus ichthyaetus* and Eurasian spoonbills *Platalea leucorodia*, that are rarely found on outbreak lakes elsewhere in Mongolia), but without further information this is difficult to surmise. Although there were large numbers of birds recorded during the surveys at Uuvs Nuur in June 2009, the estimated total density (187 birds km^−2^) was lower than recorded at all other outbreak investigations (Table S4). Outbreaks initiated in late July coincide with the highest numbers of birds on Mongolian lakes (Figure 3). For instance, counts on Erhel Nuur during the outbreak in July 2009 reached 12,278 birds, equating to a density of 662 birds km^−2^. At this time many species, particularly ruddy shelducks, congregate in large numbers to molt, at which time they are completely flightless.

The observation that no Mongolian outbreaks have arisen in the months of August, September or October is interesting, as these months are traditionally when LPAIV subtypes reach their peak prevalence in waterfowl populations in Europe and North America [46], [47]. These peaks of LPAIV subtypes are attributed to greater densities of susceptible hosts as populations are swollen by numbers of immunologically naïve juvenile birds, which congregate prior to the southward migration period. The lack of Mongolian HPAIV outbreaks detected during the late summer period suggests that the epidemiology of the virus may be quite different from natural circulation of LPAIV subtypes, which are typically less pathogenic for wild birds, although the possibility that outbreaks have been missed cannot be excluded entirely.

In many cases the numbers of sick or dead birds observed during outbreaks was unremarkable, exceeding 100 birds in only four outbreaks, with as few as a single bird involved in others. These numbers contrast with figures reported in China (Table S7), where mortalities numbering in the hundreds is more usual, with over 6,000 dead birds reported during the 2005 outbreak at Qinghai. This disparity could be related to smaller numbers of birds on Mongolian lakes, and although total population estimates are not available in many of the Chinese outbreaks, we feel that this is unlikely. For instance, over 12,000 live birds were observed during the outbreak on Erhel Nuur in 2009, yet only three dead Mongolian gulls and a single ruddy shelduck were found despite extensive searching over several days. These birds were all flightless at the time of death, indicating that they had contracted the virus locally. A review of surveillance data from Sweden and Denmark in 2006 also found that many outbreaks were associated with unspectacular levels of mortality, with 75% detected through singleton dead birds [48]. Although it is acknowledged that passive surveillance systems that investigate individual bird deaths are economically demanding, Mongolian findings indicate that not doing so will negatively influence detection sensitivity.

Although presence or abundance of a species at an outbreak site does not directly implicate it in the transmission of HPAIV, it may be instructive to look for commonalities across outbreak locations. Bird counts and species densities were assessed during five of the eleven outbreaks (Table S4) and covered all of the lakes at which outbreaks took place except Khunt Nuur. For this site, a census was made on 3 May 2007 and was considered indicative of birds present during the 4 May 2006 outbreak at that location. Only eight species were found on all of the outbreak lakes (demoiselle crane, whooper swan, ruddy shelduck, common goldeneye, common pochard, northern pintail, Mongolian gull and pied avocet). Ruddy shelduck was by far the most numerous species observed, with almost twice as many present as the next most abundant species on outbreak lakes. It has been postulated that bar-headed geese may play a role in the transmission of HPAIV across large distances in Asia [49]–[51], but this would not appear to be the case in all of the Mongolian outbreaks. Although bar-headed geese are common in the north-central region, they are much less common in western Mongolia, and none were observed in the vicinity of the 2009 outbreak on Uuvs Nuur, or the 2010 outbreak in Ganga Nuur Nature Reserve.

In conclusion, the occurrence of outbreaks in areas deficient in domestic poultry is strong evidence that wild birds can carry HPAIV over at least moderate distances. However, failure to detect further outbreaks of clade 2.2 after June 2006, and clade 2.3.2.1 after June 2010 suggests that wild birds migrating to and from Mongolia either are not competent as indefinite reservoirs of infection or did not reach susceptible populations during our study. For wild birds it appears that passive surveillance provides a more cost effective approach to HPAIV detection than surveillance of apparently healthy individuals.

## Supporting Information

Table S1 Summary of samples collected during active surveillance and analysed by virus isolation. Includes number of individuals sampled each year through capture of live birds, collection of fecal samples and sampling of clinically sick and dead birds. Total numbers of birds from which samples were submitted for virus isolation by inoculation into embryonated chicken eggs (n) are given by species, along with numbers of isolates of LPAIVs (L) and HPAIVs (H). Samples listed in 2006 and 2007 represent duplicates of those testing positive by RT-PCR (Table S3). * Refers to a ruddy shelduck that tested negative for virus by virus isolation, but was positive for HPAIV H5N1 by RT-PCR.
(DOCX)

Table S2 Summary of samples collected during outbreak investigations. Includes number of individuals sampled through capture of live birds, collection of fecal samples and sampling of clinically sick and dead birds. Total numbers of birds from which samples were submitted for virus isolation by inoculation into embryonated chicken eggs (n) are given by species, along with numbers of isolates of LPAIVs (L) and HPAIVs (H). Refers to a great crested grebe that tested negative by virus isolation, but was positive for HPAIV H5N1 by RT-PCR.
(DOCX)

Table S3 Summary of samples collected during active surveillance and analysed by RT-PCR. Includes number of individuals sampled each year through capture of live birds, collection of fecal samples and sampling of clinically sick and dead birds. Total numbers of birds from which samples were submitted for RT-PCR (n) are given by species, along with numbers of influenza A viruses (AI) and influenza A viruses of subtype H5 (H5).
(DOCX)

Table S4 Bird counts and densities at Mongolian outbreak lakes. Densities are given in parentheses and are calculated based on the surface area of the lakes (Erhel Nuur 18.56 km^2^, Khunt Nuur km^2^, Doitiin Nuur km^2^, Doroo Tsagaan Nuur km^2^), or in the case of Uuvs Nuur in the length of shoreline surveyed (19.1 km) multiplied by an estimated maximum distance visible (2 km). Presence of species that could not be counted are indicated by the letter Y. All counts were made while outbreaks were in progress, with the exception of Khunt Nuur, where counts were made exactly one year after the May 2006 outbreak as an indication of birds present during a comparable season.
(DOCX)

Table S5 Summary of information published relating to wild outbreaks of highly pathogenic avian influenza virus in Mongolia.
(DOCX)

Table S6 Summary of information published relating to wild outbreaks of highly pathogenic avian influenza virus in China.
(DOCX)

Table S7 Summary of information published relating to wild outbreaks of highly pathogenic avian influenza virus in the Tyva Republic, Russian Federation.
(DOCX)
