# Correction: Highly Pathogenic Avian Influenza Virus among Wild Birds in Mongolia

**DOI:** 10.1371/annotation/2ced0370-4f34-4380-9ca9-954ba328e160

**Published:** 2012-10-31

**Authors:** Martin Gilbert, Losolmaa Jambal, William B. Karesh, Amanda Fine, Enkhtuvshin Shiilegdamba, Purevtseren Dulam, Ruuragchaa Sodnomdarjaa, Khuukhenbaatar Ganzorig, Damdinjav Batchuluun, Natsagdorj Tseveenmyadag, Purevsuren Bolortuya, Carol J. Cardona, Connie Y. H. Leung, J. S. Malik Peiris, Erica Spackman, David E. Swayne, Damien O. Joly

There were errors in the Funding section. The correct funding information is as follows: Support for this project was provided by the National Institute of Allergy and Infectious Disease, National Institutes of Health, Department of Health and Human Services (Contracts HHSN266200700007C, HHSN266200700009C, and HHSN266200700005C), the World Bank, and the Food and Agriculture Organization of the United Nations. This work was made possible through support provided by the Office of Health, Infectious Disease and Nutrition, Bureau for Global Health, U.S. Agency for International Development and Wildlife Conservation Society, under the terms of Leader Award No.LAG-A-00-99-00047-00, Cooperative Agreement: GHS-A-00-06-00005. The opinions expressed herein are those of the author(s) and do not necessarily reflect the views of the U.S. Agency for International Development or Wildlife Conservation Society. DOJ was supported by generous funding from the Dunemere private foundation. The funders had no role in study design, data collection and analysis, decision to publish, or preparation of the manuscript.

