# Supplementary material for: Highly Pathogenic Avian Influenza Virus among Wild Birds in Mongolia
Source: PLoS One. 2012 Sep 11;7(9):e44097. doi: 10.1371/journal.pone.0044097 (PMC3439473; doi:10.1371/journal.pone.0044097)
Supplement: Table S1 — Summary of samples collected during active surveillance and analysed by virus isolation. Includes number of individuals sampled each year through capture of live birds, collection of fecal samples and sampling of clinically sick and dead birds. Total numbers of birds from which samples were submitted for virus isolation by inoculation into embryonated chicken eggs (n) are given by species, along with numbers of isolates of LPAIVs (L) and HPAIVs (H). Samples listed in 2006 and 2007 represent duplicates of those testing positive by RT-PCR (Table S3). * Refers to a ruddy shelduck that tested negative for virus by virus isolation, but was positive for HPAIV H5N1 by RT-PCR. (DOCX) [file pone.0044097.s001.docx]

**Online supporting information; Table S1.** Summary of samples collected during active surveillance each year through capture of live birds, collection of fecal samples and sampling of clinically sick and dead birds. Total numbers of birds from which samples were submitted for virus isolation by inoculation into embryonated chicken eggs (n) are given by species, along with numbers of isolates of LPAIVs (L) and HPAIVs (H). Samples listed in 2006 and 2007 represent duplicates of those testing positive by RT-PCR (Table S3). * Refers to a ruddy shelduck that tested negative for virus by virus isolation, but was positive for HPAIV H5N1 by RT-PCR.

|  |  | **2005** | | | **2006** | | | **2007** | | | **2008** | | | **2009** | | | **2010** | | | **2011** | | |
| --- | --- | --- | --- | --- | --- | --- | --- | --- | --- | --- | --- | --- | --- | --- | --- | --- | --- | --- | --- | --- | --- | --- |
| **Method** | **Species** | **n** | **L** | **H** | **n** | **L** | **H** | **n** | **L** | **H** | **n** | **L** | **H** | **n** | **L** | **H** | **n** | **L** | **H** | **n** | **L** | **H** |
| **Live** | **ANSERIFORMES** |  |  |  |  |  |  |  |  |  |  |  |  |  |  |  |  |  |  |  |  |  |
|  | Anas acuta | - | - | - | - | - | - | - | - | - | - | - | - | 1 | 0 | 0 | - | - | - | - | - | - |
|  | Anas clypeata | - | - | - | - | - | - | - | - | - | 1 | 0 | 0 | 1 | 0 | 0 | - | - | - | - | - | - |
|  | Anas strepera | - | - | - | - | - | - | - | - | - | - | - | - | 3 | 0 | 0 | - | - | - | - | - | - |
|  | Anser anser | - | - | - | - | - | - | - | - | - | - | - | - | 3 | 0 | 0 | - | - | - | - | - | - |
|  | Anser cygnoides | - | - | - | - | - | - | 1 | 0 | 0 | 145 | 0 | 0 | 126 | 0 | 0 | 4 | 0 | 0 | - | - | - |
|  | Anser fabalis | - | - | - | - | - | - | 4 | 0 | 0 | 67 | 0 | 0 | 1 | 0 | 0 | - | - | - | - | - | - |
|  | Anser indicus | - | - | - | - | - | - | 14 | 0 | 0 | 323 | 0 | 0 | 303 | 0 | 0 | 12 | 0 | 0 | - | - | - |
|  | Aythya fuligula | - | - | - | - | - | - | - | - | - | 2 | 0 | 0 | - | - | - | - | - | - | - | - | - |
|  | Cygnus cygnus | - | - | - | 1 | 0 | 0 | 12 | 0 | 0 | 104 | 0 | 0 | 56 | 0 | 0 | 26 | 0 | 0 | - | - | - |
|  | Melanitta fusca | - | - | - | - | - | - | - | - | - | 1 | 0 | 0 |  | - | - | - | - | - | - | - | - |
|  | Mergus merganser | - | - | - | - | - | - | - | - | - | 1 | 0 | 0 | 1 | 0 | 0 | - | - | - | - | - | - |
|  | Tadorna ferruginea | - | - | - | - | - | - | 4 | 0 | 0 | 150 | 1 | 0 | 236 | 0 | 0 | 6 | 0 | 0 | - | - | - |
|  | Actitis hypoleucos | - | - | - | - | - | - | - | - | - | 17 | 0 | 0 | - | - | - | - | - | - | - | - | - |
|  | Ardea cinerea | - | - | - | - | - | - | - | - | - | - | - | - | 15 | 0 | 0 | - | - | - | - | - | - |
|  | Calidris acuminata | - | - | - | - | - | - | - | - | - | 2 | 0 | 0 | - | - | - | - | - | - | - | - | - |
|  | **CICONIIFORMES** |  |  |  |  |  |  |  |  |  |  |  |  |  |  |  |  |  |  |  |  |  |
|  | Calidris ferruginea | - | - | - | - | - | - | - | - | - | 2 | 0 | 0 | - | - | - | - | - | - | - | - | - |
|  | Calidris minuta | - | - | - | - | - | - | - | - | - | 8 | 0 | 0 | - | - | - | - | - | - | - | - | - |
|  | Calidris ruficollis | - | - | - | - | - | - | - | - | - | 13 | 0 | 0 | - | - | - | - | - | - | - | - | - |
|  | Calidris subminuta | - | - | - | - | - | - | - | - | - | 41 | 0 | 0 | - | - | - | - | - | - | - | - | - |
|  | Calidris temminckii | - | - | - | - | - | - | - | - | - | 8 | 0 | 0 | - | - | - | - | - | - | - | - | - |
|  | Charadrius alexandrinus | - | - | - | - | - | - | - | - | - | 3 | 0 | 0 | - | - | - | - | - | - | - | - | - |
|  | Charadrius dubius | - | - | - | - | - | - | 1 | 0 | 0 | 26 | 0 | 0 | - | - | - | - | - | - | - | - | - |
|  | Chlidonias leucopterus | - | - | - | - | - | - | 1 | 0 | 0 | - | - | - | - | - | - | - | - | - | - | - | - |
|  | Gallinago gallinago | - | - | - | - | - | - | - | - | - | 1 | 0 | 0 | - | - | - | - | - | - | - | - | - |
|  | Larus mongolicus | - |  | - | - | - | - | - | - | - | 146 | 0 | 0 | 145 | 0 | 0 | - | - | - | - | - | - |
|  | Larus relictus | - | - | - | - | - | - | - | - | - | 2 | 0 | 0 | - | - | - | - | - | - | - | - | - |
|  | Chroicocephalus ridibundus | - | - | - | - | - | - | - | - | - | 1 | 0 | 0 | - | - | - | - | - | - | - | - | - |
|  | Limicola falcinellus | - | - | - | - | - | - | - | - | - | 7 | 0 | 0 | - | - | - | - | - | - | - | - | - |
|  | Limnodromus semipalmatus | - | - | - | - | - | - | - | - | - | 1 | 0 | 0 | - | - | - | - | - | - | - | - | - |
|  | Phalacrocorax carbo | - | - | - | - | - | - | 6 | 0 | 0 | 83 | 0 | 0 | - | - | - | - | - | - | - | - | - |
|  | Philomachus pugnax | - | - | - | - | - | - | - | - | - | 1 | 0 | 0 | - | - | - | - | - | - | - | - | - |
|  | Pluvialis fulva | - | - | - | - | - | - | - | - | - | 20 | 0 | 0 | - | - | - | - | - | - | - | - | - |
|  | Tringa glareola | - | - | - | - | - | - | - | - | - | 52 | 0 | 0 | - | - | - | - | - | - | - | - | - |
|  | Tringa ochropus | - | - | - | - | - | - | - | - | - | 2 | 0 | 0 | - | - | - | - | - | - | - | - | - |
|  | Tringa totanus | - | - | - | - | - | - | - | - | - | 20 | 0 | 0 | - | - | - | - | - | - | - | - | - |
|  | Vanellus vanellus | - | - | - | - | - | - | - | - | - | 3 | 0 | 0 | - | - | - | - | - | - | - | - | - |
|  | Xenus cinereus | - | - | - | - | - | - | - | - | - | 16 | 0 | 0 | - | - | - | - | - | - | - | - | - |
|  | **PASSERIFORMES** |  |  |  |  |  |  |  |  |  |  |  |  |  |  |  |  |  |  |  |  |  |
|  | Cyanopica cyanus | - | - | - | - | - | - | - | - | - | - | - | - | - | - | - | 8 | 0 | 0 | - | - | - |
|  | Emberiza cioides | - | - | - | - | - | - | - | - | - | - | - | - | - | - | - | 1 | 0 | 0 | - | - | - |
|  | Ficedula albicilla | - | - | - | - | - | - | - | - | - | - | - | - | - | - | - | 1 | 0 | 0 | - | - | - |
|  | Luscinia calliope | - | - | - | - | - | - | - | - | - | - | - | - | - | - | - | 1 | 0 | 0 | - | - | - |
|  | Luscinia svecica | - | - | - | 1 | 0 | 0 | - | - | - | - | - | - | - | - | - | - | - | - | - | - | - |
|  | Muscicapa striata | - | - | - | - | - | - | - | - | - | - | - | - | - | - | - | 13 | 0 | 0 | - | - | - |
|  | Parus cyanus | - | - | - | - | - | - | - | - | - | - | - | - | - | - | - | 4 | 0 | 0 | - | - | - |
|  | Parus major | - | - | - | - | - | - | - | - | - | - | - | - | - | - | - | 1 | 0 | 0 | - | - | - |
|  | Parus montanus | - | - | - | - | - | - | - | - | - | - | - | - | - | - | - | 7 | 0 | 0 | - | - | - |
|  | Passer montanus | - | - | - | - | - | - | - | - | - | - | - | - | - | - | - | 17 | 0 | 0 | - | - | - |
|  | Phoenicurus auroreus | - | - | - | - | - | - | - | - | - | - | - | - | - | - | - | 10 | 0 | 0 | - | - | - |
|  | Phoenicurus phoenicurus | - | - | - | - | - | - | - | - | - | - | - | - | - | - | - | 1 | 0 | 0 | - | - | - |
|  | Sylvia curruca | - | - | - | - | - | - | - | - | - | - | - | - | - | - | - | 3 | 0 | 0 | - | - | - |
|  | **PICIFORMES** |  |  |  |  |  |  |  |  |  |  |  |  |  |  |  |  |  |  |  |  |  |
|  | Dendrocopos leucotos | - | - | - | - | - | - | - | - | - | - | - | - | - | - | - | 1 | 0 | 0 | - | - | - |
|  | Dendrocopos minor | - | - | - | - | - | - | - | - | - | - | - | - | - | - | - | 1 | 0 | 0 | - | - | - |
| **Fecal** | **ANSERIFORMES** |  |  |  |  |  |  |  |  |  |  |  |  |  |  |  |  |  |  |  |  |  |
|  | Anser indicus | 97 | 1 | 0 | 5 | 0 | 0 | - | - | - | - | - | - | - | - | - | - | - | - | - | - | - |
|  | Anser spp. | - | - | - | - | - | - | - | - | - | - | - | - | 120 | 0 | 0 | - | - | - | - | - | - |
|  | Cygnus cygnus | 73 | 0 | 0 | 10 | 0 | 0 | - | - | - | - | - | - | - | - | - | - | - | - | - | - | - |
|  | Cygnus spp. | - | - | - | 30 | 0 | 0 | - | - | - | - | - | - | - | - | - | - | - | - | - | - | - |
|  | Mixed duck spp. | - | - | - | - | - | - | - | - | - | - | - | - | 480 | 1 | 0 | 1521 | 23 | 0 | 2400 | 26 | 0 |
|  | Anas crecca | - | - | - | 5 | 0 | 0 | - | - | - | - | - | - | - | - | - | - | - | - | - | - | - |
|  | Aythya ferina | - | - | - | 10 | 0 | 0 | - | - | - | - | - | - | - | - | - | - | - | - | - | - | - |
|  | Rhodonessa rufina | - | - | - | 15 | 1 | 0 | - | - | - | - | - | - | 120 | 1 | 0 | - | - | - | - | - | - |
|  | Tadorna ferruginea | 243 | 1 | 0 | 15 | 0 | 0 | - | - | - | - | - | - | - | - | - | - | - | - | - | - | - |
|  | Tadorna tadorna | - | - | - | 5 | 0 | 0 | - | - | - | - | - | - | - | - | - | - | - | - | - | - | - |
|  | **CICONIIFORMES** |  |  |  |  |  |  |  |  |  |  |  |  |  |  |  |  |  |  |  |  |  |
|  | Larus mongolicus | - | - | - | 25 | 0 | 0 | - | - | - | - | - | - | 100 | 0 | 0 | - | - | - | - | - | - |
|  | Chroicocephalus ridibundus | 35 | 0 | 0 | 35 | 2 | 0 | - | - | - | - | - | - | - | - | - | - | - | - | - | - | - |
|  | Larus spp. | - | - | - | - | - | - | - | - | - | - | - | - | 40 | 0 | 0 | - | - | - | - | - | - |
|  | Phalacrocorax carbo | - | - | - | 20 | 0 | 0 | - | - | - | - | - | - | - | - | - | - | - | - | - | - | - |
|  | Vanellus vanellus | - | - | - | 5 | 0 | 0 | - | - | - | - | - | - | - | - | - | - | - | - | - | - | - |
| **Sick/ Dead** | **ANSERIFORMES** |  |  |  |  |  |  |  |  |  |  |  |  |  |  |  |  |  |  |  |  |  |
|  | Cygnus columbianus | - | - | - | - | - | - | - | - | - | - | - | - | 1 | 0 | 0 | - | - | - | - | - | - |
|  | Cygnus cygnus | 1 | 0 | 0 | - | - | - | - | - | - | 1 | 0 | 0 | 1 | 0 | 0 | - | - | - | - | - | - |
|  | Tadorna ferruginea | - | - | - | - | - | - | - | - | - | - | - | - | 1 | 0 | * | - | - | - | - | - | - |
|  | Bucephala clangula | - | - | - | 1 | 0 | 0 | - | - | - | - | - | - | - | - | - | - | - | - | - | - | - |
|  | **CICONIIFORMES** |  |  |  |  |  |  |  |  |  |  |  |  |  |  |  |  |  |  |  |  |  |
|  | Actitis hypoleucos | - | - | - | - | - | - | - | - | - | - | - | - | 2 | 0 | 0 | - | - | - | - | - | - |
|  | Larus mongolicus | - | - | - | - | - | - | 5 | 2 | 0 | 48 | 0 | 0 | 3 | 0 | 3 | 1 | 0 | 0 | - | - | - |
|  | Chroicocephalus ridibundus | - | - | - | - | - | - | - | - | - | 1 | 0 | 0 | 1 | 0 | 0 | - | - | - | - | - | - |
|  | Phalacrocorax carbo | - | - |  | - | - | - | - | - | - | 2 | 0 | 0 | 1 | 0 | 0 | - | - | - | - | - | - |
|  | Podiceps cristatus | - | - | - | - | - | - | - | - | - | - | - | - | 1 | 0 | 0 | - | - | - | - | - | - |
|  | Stercorarius longicaudus | - | - | - | - | - | - | - | - | - | 1 | 0 | 0 | - | - | - | - | - | - | - | - | - |
|  | Vanellus vanellus | - | - | - | 1 | 0 | 0 | - | - | - | - | - | - | - | - | - | - | - | - | - | - | - |
|  | **PASSERIFORMES** |  |  |  |  |  |  |  |  |  |  |  |  |  |  |  |  |  |  |  |  |  |
|  | Calandrella cheleensis | - | - | - | 1 | 0 | 0 | - | - | - | - | - | - | - | - | - | - | - | - | - | - | - |
|  | Corvus corax | - | - | - | - | - | - | - | - | - | - | - | - | 3 | 0 | 0 | - | - | - | - | - | - |
|  | **TOTAL** | **449** | **2** | **0** | **185** | **3** | **0** | **48** | **2** | **0** | **1322** | **1** | **0** | **1765** | **2** | **3** | **1639** | **23** | **0** | **2400** | **26** | **0** |
