# Supplementary material for: Highly Pathogenic Avian Influenza Virus among Wild Birds in Mongolia
Source: PLoS One. 2012 Sep 11;7(9):e44097. doi: 10.1371/journal.pone.0044097 (PMC3439473; doi:10.1371/journal.pone.0044097)
Supplement: Table S2 — Summary of samples collected during outbreak investigations. Includes number of individuals sampled through capture of live birds, collection of fecal samples and sampling of clinically sick and dead birds. Total numbers of birds from which samples were submitted for virus isolation by inoculation into embryonated chicken eggs (n) are given by species, along with numbers of isolates of LPAIVs (L) and HPAIVs (H). Refers to a great crested grebe that tested negative by virus isolation, but was positive for HPAIV H5N1 by RT-PCR. (DOCX) [file pone.0044097.s002.docx]

**Online supporting information; Table S2.** Summary of samples collected during outbreak investigations each year through capture of live birds, collection of fecal samples and sampling of clinically sick and dead birds. Total numbers of birds from which samples were submitted for virus isolation by inoculation into embryonated chicken eggs (n) are given by species, along with numbers of isolates of LPAIVs (L) and HPAIVs (H). Refers to a great crested grebe that tested negative for virus by virus isolation, but was positive for HPAIV H5N1 by RT-PCR

|  |  | **2005** | | | **2006** | | | **2007** | | | **2008** | | | **2009** | | | **2010** | | | **2011** | | |
| --- | --- | --- | --- | --- | --- | --- | --- | --- | --- | --- | --- | --- | --- | --- | --- | --- | --- | --- | --- | --- | --- | --- |
| **Method** | **Species** | **n** | **L** | **H** | **n** | **L** | **H** | **n** | **L** | **H** | **n** | **L** | **H** | **n** | **L** | **H** | **n** | **L** | **H** | **n** | **L** | **H** |
| **Live bird** | **ANSERIFORMES** |  |  |  |  |  |  |  |  |  |  |  |  |  |  |  |  |  |  |  |  |  |
|  | Anas penelope | 1 | 0 | 0 | - | - | - | - | - | - | - | - | - | - | - | - | - | - | - | - | - | - |
|  | Anser indicus | - | - | - | - | - | - | - | - | - | - | - | - | 9 | 0 | 0 | - | - | - | - | - | - |
|  | Cygnus cygnus | - | - | - | - | - | - | - | - | - | - | - | - | 1 | 0 | 0 | - | - | - | - | - | - |
|  | Tadorna ferruginea | - | - | - | - | - | - | - | - | - | - | - | - | 81 | 0 | 0 | - | - | - | - | - | - |
|  | **CICONIIFORMES** |  |  |  |  |  |  |  |  |  |  |  |  |  |  |  |  |  |  |  |  |  |
|  | Larus mongolicus | 1 | 0 | 0 | - | - | - | - | - | - | - | - | - | - | - | - | - | - | - | - | - | - |
| **Fecal** | **ANSERIFORMES** |  |  |  |  |  |  |  |  |  |  |  |  |  |  |  |  |  |  |  |  |  |
|  | Anser indicus | 34 | 0 | 0 | - | - | - | - | - | - | - | - | - | - | - | - | - | - | - | - | - | - |
|  | Cygnus cygnus | 18 | 1 | 0 | - | - | - | - | - | - | - | - | - | - | - | - | - | - | - | - | - | - |
|  | Mixed duck spp. | - | - | - | - | - | - | - | - | - | - | - | - | 141 | 18 | 0 | 151 | 0 | 0 | - | - | - |
|  | Tadorna ferruginea | 360 | 1 | 0 | - | - | - | - | - | - | - | - | - | - | - | - | - | - | - | - | - | - |
|  | **CICONIIFORMES** |  |  |  |  |  |  |  |  |  |  |  |  |  |  |  |  |  |  |  |  |  |
|  | Larus mongolicus | 19 | 0 | 0 | - | - | - | - | - | - | - | - | - | - | - | - | - | - | - | - | - | - |
| **Sick/ dead** | **ANSERIFORMES** |  |  |  |  |  |  |  |  |  |  |  |  |  |  |  |  |  |  |  |  |  |
|  | Anser indicus | 3 | 0 | 0 | - | - | - | - | - | - | - | - | - | 2 | 0 | 1 | - | - | - | - | - | - |
|  | Cygnus cygnus | 1 | 0 | 1 | - | - | - | - | - | - | - | - | - | - | - | - | - | - | - | - | - | - |
|  | Cygnus columbianus | - | - | - | - | - | - | - | - | - | - | - | - |  | - | - | 1 | 0 | 1 | - | - | - |
|  | Tadorna ferruginea | - | - | - | - | - | - | - | - | - | - | - | - | 2 | 0 | 2 | - | - | - | - | - | - |
|  | **CICONIIFORMES** |  |  |  |  |  |  |  |  |  |  |  |  |  |  |  |  |  |  |  |  |  |
|  | Larus mongolicus | 2 | 0 | 0 | - | - | - | - | - | - | - | - | - | - | - | - | - | - | - | - | - | - |
|  | Larus ichthyaetus | - | - | - | - | - | - | - | - | - | - | - | - | 1 | 0 | 0 | - | - | - | - | - | - |
|  | Chroicocephalus ridibundus | - | - | - | - | - | - | - | - | - | - | - | - | 2 | 0 | 0 | - | - | - | - | - | - |
|  | Podiceps cristatus | - | - | - | - | - | - | - | - | - | - | - | - | 1 | 0 | * | - | - | - | - | - | - |
|  | **TOTAL** | **439** | **2** | **1** | **0** | **0** | **0** | **0** | **0** | **0** | **0** | **0** | **0** | **240** | **18** | **3** | **152** | **0** | **1** | **0** | **0** | **0** |
