# Supplementary material for: Highly Pathogenic Avian Influenza Virus among Wild Birds in Mongolia
Source: PLoS One. 2012 Sep 11;7(9):e44097. doi: 10.1371/journal.pone.0044097 (PMC3439473; doi:10.1371/journal.pone.0044097)
Supplement: Table S3 — Summary of samples collected during active surveillance and analysed by RT-PCR. Includes number of individuals sampled each year through capture of live birds, collection of fecal samples and sampling of clinically sick and dead birds. Total numbers of birds from which samples were submitted for RT-PCR (n) are given by species, along with numbers of influenza A viruses (AI) and influenza A viruses of subtype H5 (H5). (DOCX) [file pone.0044097.s003.docx]

**Online supporting information; Table S3.** Summary of samples collected during active surveillance from 2006 to 2008 through capture of live birds, collection of fecal samples and sampling of clinically sick and dead birds. Total numbers of birds from which samples were submitted for RT-PCR (n) are given by species, along with numbers of influenza A viruses (AI) and influenza A viruses of subtype H5 (H5).

|  |  | **2006** | | | **2007** | | | **2008** | | | |  |
| --- | --- | --- | --- | --- | --- | --- | --- | --- | --- | --- | --- | --- |
| **Method** | **Species** | **n** | **AI** | **H5** | **n** | **AI** | **H5** | **n** | **AI** | **H5** | |  |
| **Live bird** | **ANSERIFORMES** |  |  |  |  |  |  |  |  |  | |  |
|  | Anas clypeata | - | - | - | - | - | - | 1 | 0 | 0 | |  |
|  | Anas crecca | - | - | - | 1 | 0 | 0 | - | - | - | |  |
|  | Anas penelope | - | - | - | 2 | 0 | 0 | - | - | - | |  |
|  | Anser anser | - | - | - | 1 | 0 | 0 | - | - | - | |  |
|  | Anser cygnoides | - | - | - | 17 | 1 | 0 | 151 | 28 | 0 | |  |
|  | Anser fabalis | - | - | - | 21 | 4 | 0 | 69 | 3 | 0 | |  |
|  | Anser indicus | - | - | - | 118 | 14 | 0 | 323 | 15 | 0 | |  |
|  | Aythya fuligula | - | - | - | - | - | - | 2 | 0 | 0 | |  |
|  | Cygnus cygnus | 8 | 1 | 0 | 121 | 12 | 0 | 104 | 10 | 0 | |  |
|  | Melanitta fusca | - | - | - | 1 | 0 | 0 | 1 | 0 | 0 | |  |
|  | Mergus merganser | - | - | - | - | - | - | 1 | 0 | 0 | |  |
|  | Tadorna ferruginea | - | - | - | 33 | 4 | 0 | 149 | 12 | 0 | |  |
|  | **CICONIIFORMES** |  |  |  |  |  |  |  |  |  | |  |
|  | Actitis hypoleucos | - | - | - | - | - | - | 18 | 1 | 0 | |  |
|  | Arenaria interpres | - | - | - | 2 | 0 | 0 | - | - | - | |  |
|  | Calidris acuminata | - | - | - | - | - | - | 2 | 1 | 0 | |  |
|  | Calidris ferruginea | - | - | - | 7 | 0 | 0 | 2 | 0 | 0 | |  |
|  | Calidris minuta | 2 | 0 | 0 | 1 | 0 | 0 | 8 | 1 | 0 | |  |
|  | Calidris ruficollis | - | - | - | - | - | - | 16 | 3 | 1 | |  |
|  | Calidris subminuta | - | - | - | 1 | 0 | 0 | 46 | 5 | 0 | |  |
|  | Calidris temminckii | 3 | 0 | 0 | - | - | - | 9 | 1 | 0 | |  |
|  | Charadrius alexandrinus | 2 | 0 | 0 | 3 | 0 | 0 | 3 | 0 | 0 | |  |
|  | Charadrius dubius | - | - | - | 2 | 1 | 0 | 29 | 3 | 0 | |  |
|  | Chlidonias leucopterus | - | - | - | 4 | 1 | 0 | - | - | - | |  |
|  | Gallinago gallinago | - | - | - | - | - | - | 2 | 1 | 0 | |  |
|  | Gallinago stenura | - | - | - | 1 | 0 | 0 | - | - | - | |  |
|  | Gavia arctica | - | - | - | 1 | 0 | 0 | - | - | - | |  |
|  | Larus mongolicus | - | - | - | 3 | 0 | 0 | 146 | 6 | 0 | |  |
|  | Larus relictus | - | - | - | - | - | - | 2 | 0 | 0 | |  |
|  | Chroicocephalus ridibundus | - | - | - | - | - | - | 1 | 1 | 0 | |  |
|  | Limicola falcinellus | 1 | 0 | 0 | - | - | - | 7 | 0 | 0 | |  |
|  | Limnodromus semipalmatus | - | - | - | - | - | - | 1 | 0 | 0 | |  |
|  | Phalacrocorax carbo | - | - | - | 96 | 6 | 0 | 105 | 22 | 0 | |  |
|  | Philomachus pugnax | - | - | - | 3 | 0 | 0 | 1 | 0 | 0 | |  |
|  | Pluvialis fulva | - | - | - | 14 | 0 | 0 | 22 | 2 | 0 | |  |
|  | Podiceps cristatus | - | - | - | 1 | 0 | 0 | - | - | - | |  |
|  | Sterna hirundo | 1 | 0 | 0 | 2 | 0 | 0 | - | - | - | |  |
|  | Tringa erythropus | - | - | - | 1 | 0 | 0 | - | - | - | |  |
|  | Tringa glareola | - | - | - | 3 | 0 | 0 | 53 | 3 | 0 | |  |
|  | Tringa ochropus | - | - | - | - | - | - | 2 | 1 | 0 | |  |
|  | Tringa totanus | - | - | - | 3 | 0 | 0 | 23 | 3 | 0 | |  |
|  | Vanellus vanellus | - | - | - | - | - | - | 3 | 0 | 0 | |  |
|  | Xenus cinereus | - | - | - | - | - | - | 18 | 2 | 0 | |  |
|  | **PASSERIFORMES** |  |  |  |  |  |  |  |  |  | |  |
|  | Luscinia svecica | 3 | 1 | 0 | - | - | - | - | - | - | |  |
|  | Phylloscopus collybita tristis | 3 | 0 | 0 | - | - | - | - | - | - | |  |
|  | Sylvia curruca | 2 | 0 | 0 | - | - | - | - | - | - | |  |
| **Fecal** | **ANSERIFORMES** |  |  |  |  |  |  |  |  |  | |  |
|  | Anas crecca | 120 | 5 | 0 | - | - | - | - | - | - | |  |
|  | Anser cygnoides | 45 | 0 | 0 | - | - | - | - | - | - | |  |
|  | Anser indicus | 70 | 5 | 0 | - | - | - | - | - | - | |  |
|  | Aythya ferina | 130 | 10 | 0 | - | - | - | - | - | - | |  |
|  | Cygnus cygnus | 45 | 10 | 0 | - | - | - | - | - | - | |  |
|  | Cygnus spp. | 135 | 30 | 0 | - | - | - | - | - | - | |  |
|  | Rhodonessa rufina | 195 | 15 | 1 | - | - | - | - | - | - | |  |
|  | Tadorna ferruginea | 480 | 15 | 0 | - | - | - | - | - | - | |  |
|  | Tadorna tadorna | 45 | 5 | 0 | - | - | - | - | - | - | |  |
|  | **CICONIIFORMES** |  |  |  |  |  |  |  |  |  | |  |
|  | Larus mongolicus | 305 | 25 | 0 | - | - | - | - | - | - | |  |
|  | Chroicocephalus ridibundus | 310 | 35 | 0 | - | - | - | - | - | - | |  |
|  | Phalacrocorax carbo | 630 | 20 | 0 | - | - | - | - | - | - | |  |
|  | Vanellus vanellus | 100 | 5 | 0 | - | - | - | - | - | - | |  |
|  | **GRUIFORMES** |  |  |  |  |  |  |  |  |  | |  |
|  | Grus virgo | 15 | 0 | 0 | - | - | - | - | - | - | |  |
|  | **PASSERIFORMES** |  |  |  |  |  |  |  |  |  | |  |
|  | Passer montanus | 1 | 0 | 0 | - | - | - | - | - | - | |  |
| **Sick/ dead** | **ANSERIFORMES** |  |  |  |  |  |  |  |  |  | |  |
|  | Anas crecca | - | - | - | 1 | 0 | 0 | - | - | - | |  |
|  | Anser cygnoides | 6 | 0 | 0 | - | - | - | - | - | - | |  |
|  | Anser indicus | - | - | - | 1 | 0 | 0 | - | - | - | |  |
|  | Aythya fuligula | 1 | 0 | 0 | - | - | - | - | - | - | |  |
|  | Bucephala clangula | 1 | 1 | 0 | - | - | - | - | - | - | |  |
|  | Cygnus columbianus | 1 | 0 | 0 | - | - | - | - | - | - | |  |
|  | Cygnus cygnus | - | - | - | - | - | - | 1 | 0 | 0 | |  |
|  | Melanitta fusca | - | - | - | 1 | 0 | 0 | - | - | - | |  |
|  | Tadorna ferruginea | - | - | - | 1 | 0 | 0 | - | - | - | |  |
|  | Tadorna tadorna | 1 | 0 | 0 | - | - | - | - | - | - | |  |
|  | **CICONIIFORMES** |  |  |  |  |  |  |  |  |  | |  |
|  | Larus mongolicus | 1 | 0 | 0 | 40 | 5 | 0 | 49 | 3 | 0 | |  |
|  | Chroicocephalus ridibundus | 2 | 0 | 0 | - | - | - | 1 | 0 | 0 | |  |
|  | Phalacrocorax carbo | 10 | 0 | 0 | - | - | - | 5 | 3 | 0 | |  |
|  | Stercorarius longicaudus | - | - | - | - | - | - | 1 | 0 | 0 | |  |
|  | Tringa glareola | 1 | 0 | 0 | - | - | - | - | - | - | |  |
|  | Vanellus vanellus | 1 | 1 | 0 | - | - | - | - | - | - | |  |
|  | **FALCONIFORMES** |  |  |  |  |  |  |  |  |  | |  |
|  | Aquila chrysaetos | 1 | 0 | 0 | - | - | - | - | - | - | |  |
|  | **PASSERIFORMES** |  |  |  |  |  |  |  |  |  | |  |
|  | Calandrella cheleensis | 1 | 1 | 0 | - | - | - | - | - | - | |  |
|  | **UPUPIFORMES** |  |  |  |  |  |  |  |  |  | |  |
|  | Upupa epops | - | - | - | 1 | 0 | 0 | - | - | - | |  |
|  | **TOTAL** | **2678** | **185** | **1** | **508** | **48** | **0** | **1377** | **130** | | **1** | |
