# Supplementary material for: Highly Pathogenic Avian Influenza Virus among Wild Birds in Mongolia
Source: PLoS One. 2012 Sep 11;7(9):e44097. doi: 10.1371/journal.pone.0044097 (PMC3439473; doi:10.1371/journal.pone.0044097)
Supplement: Table S4 — Bird counts and densities at Mongolian outbreak lakes. Densities are given in parentheses and are calculated based on the surface area of the lakes (Erhel Nuur 18.56 km2, Khunt Nuur km2, Doitiin Nuur km2, Doroo Tsagaan Nuur km2), or in the case of Uuvs Nuur in the length of shoreline surveyed (19.1 km) multiplied by an estimated maximum distance visible (2 km). Presence of species that could not be counted are indicated by the letter Y. All counts were made while outbreaks were in progress, with the exception of Khunt Nuur, where counts were made exactly one year after the May 2006 outbreak as an indication of birds present during a comparable season. (DOCX) [file pone.0044097.s004.docx]

**Online supporting information; Table S4.** Bird counts and densities (given in parentheses as birds/km^2^) at Mongolian outbreak lakes. Densities are calculated based on the surface area of the lakes (Erhel Nuur 18.56 km^2^, Khunt Nuur km^2^, Doitiin Nuur km^2^, Doroo Tsagaan Nuur km^2^), or in the case of Uuvs Nuur in the length of shoreline surveyed (19.1 km) multiplied by an estimated maximum distance visible (2 km). Presence of species that could not be counted a Leannere indicated by the letter Y. All counts were made while outbreaks were in progress, with the exception of Khunt Nuur, where counts were made exactly one year after the May 2006 outbreak as an indication of birds present during a comparable season.

| **Species common name** | **Species scientific name** | **Erhel Nuur,**  **August 2005** | **Khunt Nuur,**  **May 2007** | **Doitiin Nuur,**  **June 2009** | **Uuvs Nuur,**  **June 2009** | **Erhel Nuur,**  **July 2009** | **Doroo Tsagaan Nuur, August 2009** | **Ganga Nuur,**  **May 2010** |
| --- | --- | --- | --- | --- | --- | --- | --- | --- |
| Black-necked grebe | *Podiceps nigricollis* | 95 (5.1) | 2 (2.1) | - | - | 144 (7.8) | 224 (28.7) | - |
| Slavonian grebe | *Podiceps auritus* | 2 (0.1) | - | - | - | - | 3 (0.4) | 1 (0.1) |
| Great-crested grebe | *Podiceps cristatus* | - | - | - | 65 (1.7) | - | 1 (0.1) | - |
| Great cormorant | *Phalacrocorax carbo* | - | 3 (3.2) | - | 1,980 (51.8) | - | - | - |
| Common coot | *Fulica atra* | - | - | 8 (5.8) | 15 (0.4) | - | - | - |
| Demoiselle crane | *Grus virgo* | 55 (3.0) | 7 (7.4) | 16 (11.7) | 5 (0.1) | 8 (0.4) | 3 (0.4) | 16 (1.6) |
| Common crane | *Grus grus* | - | 10 (10.5) | - | 3 (0.1) | - | - | - |
| Great bustard | *Otis tarda* | - | - | - | 1 (0) | - | - | - |
| Eurasian spoonbill | *Platalea leucorodia* | 35 (1.9) | 1 (1.1) | - | 56 (1.5) | - | - | - |
| Grey heron | *Ardea cinerea* | 2 (0.1) | 129 (135.8) | 1 (0.7) | 44 (1.2) | 4 (0.2) | 1 (0.1) | - |
| Great white egret | *Egretta alba* | - | - | - | 5 (0.1) | - | - | - |
| White-tailed eagle | *Haliaeetus albicilla* | - | 1 (1.1) | - | 2 (0.1) | 1 (0.1) | 2 (0.3) | - |
| Cinereous vulture | *Aegypius monachus* | - | 2 (2.1) | - | - | 20 (1.1) | 5 (0.6) | - |
| Steppe eagle | *Aquila nipalensis* | - | - | 2 (1.5) | - | - | 1 (0.1) | - |
| Golden eagle | *Aquila chrysaetos* | - | 1 (1.1) | - | - | 1 (0.1) | - | - |
| Aquila sp. | *Aquila sp.* | 2 (0.1) | - | - | - | - | - | - |
| Amur falcon | *Falco amurensis* | - | - | - | - | - | 1 (0.1) | 1 (0.1) |
| Merlin | *Falco columbarius* | - | - | - | - | 1 (0.1) | - | - |
| Upland buzzard | *Buteo hemilasius* | - | - | 3 (2.2) | 1 (0) | - | - | - |
| Black kite | *Milvus migrans* | 1 (0.1) | - | - | 10 (0.3) | 6 (0.3) | 1 (0.1) | 2 (0.2) |
| Eurasian hobby | *Falco subuteo* | 1 (0.1) | - | - | - | - | - | - |
| Saker falcon | *Falco cherrug* | 1 (0.1) | - | - | - | - | - | - |
| Common kestrel | *Falco tinnunculus* | - | - | - | 6 (0.2) | - | 5 (0.6) | 1 (0.1) |
| Lesser kestrel | *Falco naumanni* | - | - | - | 10 (0.3) | - | - | - |
| Pallas' sandgrouse | *Syrrhaptes paradoxus* | - | - | - | 6 (0.2) | - | - | 19 (1.9) |
| Whooper swan | *Cygnus cygnus* | 48 (2.6) | 2 (2.1) | 54 (39.4) | 3 (0.1) | 22 (1.2) | 8 (1.0) | 84 (8.3) |
| Tundra swan | *Cygnus columbianus* | - | 2 (2.1) | - | - | 1 (0.1) | - | 201 (19.8) |
| Bar-headed goose | *Anser indicus* | 165 (8.9) | 80 (84.2) | 20 (14.6) | - | 222 (12) | 124 (15.9) | - |
| Swan goose | *Anser cygnoides* | 7 (0.4) | 6 (6.3) | 80 (58.4) | 12 (0.3) | 4 (0.2) | - | - |
| Greylag goose | *Anser anser* | - | - | - | 100 (2.6) | - | - | - |
| Bean goose | *Anser fabalis* | - | - | - | - | 1 (0.1) | - | 1 (0.1) |
| Common shelduck | *Tadorna tadorna* | 4 (0.2) | 9 (9.5) | 10 (7.3) | 7 (0.2) | 20 (1.1) | - | 483 (47.7) |
| Ruddy shelduck | *Tadorna ferruginea* | 2,943 (158.6) | 37 (38.9) | 40 (29.2) | 36 (0.9) | 5,308 (286.0) | 1,577 (202.2) | 452 (44.6) |
| Common goldeneye | *Bucephala clangula* | 1812 (97.6) | 8 (8.4) | 80 (58.4) | 30 (0.8) | 3,332 (179.5) | 803 (102.9) | 8 (0.8) |
| Common pochard | *Aythya ferina* | 404 (21.8) | 50 (52.6) | 20 (14.6) | 200 (5.2) | 2,284 (123.1) | 194 (24.9) | 71 (7.0) |
| Tufted duck | *Aythya fuligula* | 52 (2.8) | 180 (189.5) | 40 (29.2) | - | 211 (11.4) | 622 (79.7) | 148 (14.6) |
| Mallard | *Anas platyrhynchos* | 40 (2.2) | 6 (6.3) | - | 8 (0.2) | 11 (0.6) | 4 (0.5) | 21 (2.1) |
| Common teal | *Anas crecca* | 3 (0.2) | 27 (28.4) | - | 250 (6.5) | 11 (0.6) | 8 (1.0) | 66 (6.5) |
| Northern pintail | *Anas acuta* | 5 (0.3) | 96 (101.1) | 16 (11.7) | Y | 12 (0.6) | 4 (0.5) | 42 (4.1) |
| Northern shoveler | *Anas clypeata* | 3 (0.2) | 18 (18.9) | 10 (7.3) | - | 17 (0.9) | 8 (1.0) | 38 (3.8) |
| Falcated Duck | *Anas falcata* | - | - | - | - | - | - | 3 (0.3) |
| Spotbilled duck | *Anas poecilorhyncha* | - | - | 10 (7.3) | - | - | - | - |
| Garganey | *Anas querquedula* | - | - | 12 (8.8) | 15 (0.4) | - | - | 1 (0.1) |
| Eurasian wigeon | *Anas penelope* | 13 (0.7) | 40 (42.1) | - | 37 (1) | 52 (2.8) | 13 (1.7) | 234 (23.1) |
| Gadwall | *Anas strepera* | 2 (0.1) | 79 (83.2) | 44 (32.1) | 7 (0.2) | 5 (0.3) | - | 31 (3.1) |
| Smew | *Mergellus albellus* | 1 (0.1) | 2 (2.1) | - | - | - | 1 (0.1) | - |
| White-winged scotor | *Melanitta deglandi stejnegeri* | 3 (0.2) | 7 (7.4) | - | - | 24 (1.3) | 64 (8.2) | 9 (0.9) |
| Red-crested pochard | *Rhodonessa rufina* | - | 4 (4.2) | - | 207 (5.4) | - | - | - |
| Pallas’s gull | *Larus ichthyaetus* | - | - | - | 2505 (65.6) | - | - | - |
| Black-headed gull | *Chroicocephalus ridibundus* | 4 (0.2) | 1 (1.1) | - | 840 (22) | 94 (5.1) | 23 (2.9) | 3 (0.3) |
| Mongolian gull | *Larus mongolicus* | 557 (30.0) | 128 (134.7) | 2 (1.5) | 160 (4.2) | 377 (20.3) | 401 (51.4) | 86 (8.5) |
| Common gull | *Larus canus* | - | - | 1 (0.7) | - | - | - | - |
| Little gull | *Larus minutus* | 1 (0.1) | - | - | - | - | - | - |
| Caspian tern | *Sterna caspia* | - | - | - | 2 (0.1) | - | - | - |
| Gull-billed tern | *Gelochelidon nilotica* | - | - | - | - | - | - | 5 (0.5) |
| Common tern | *Sterna hirundo* | - | - | - | 275 (7.2) | - | - | - |
| Pacific golden plover | *Pluvialis fulva* | - | - | - | 1 (0) | 1 (0.1) | - | - |
| Mongolian plover | *Charadrius mongolus* | - | - | - | 4 (0.1) | - | - | - |
| Ruddy turnstone | *Arenaria interpres* | - | - | - | - | 1 (0.1) | 1 (0.1) | - |
| Curlew sandpiper | *Calidris ferruginea* | - | - | - | - | 16 (0.9) | 1 (0.1) | - |
| Eurasian curlew | *Numenius arquata* | 50 (2.7) | Y | - | 2 (0.1) | - | - | - |
| Whimbrel | *Numenius phaeopus* | - | - | - | - | 1 (0.1) | - | - |
| Little curlew | *Numenius minutus* | - | - | - | - | - | - | 1 (0.1) |
| Black-tailed godwit | *Limosa limosa* | 2 (0.1) | - | 10 (7.3) | - | - | - | 7 (0.7) |
| Little stint | *Calidris minuta* | - | - | - | - | - | - | 5 (0.5) |
| Temmink's stint | *Calidris temminkii* | Y | - | - | - | 5 (0.3) | 1 (0.1) | 160 (15.8) |
| Sanderling | *Calidris alba* | - | - | 1 (0.7) | - | - | - | - |
| Sharp-tailed sandpiper | *Calidris acuminata* | - | - | 10 (7.3) | - | - | - | - |
| Red-necked stint | *Calidris rufficollis* | 2 (0.1) | - | - | - | 1 (0.1) | - | 2 (0.2) |
| Long-toed stint | *Calidris subminuta* | Y | - | - | - | 6 (0.3) | - | - |
| Green sandpiper | *Tringa ochropus* | Y | 2 (2.1) | 2 (1.5) | - | - | Y | 2 (0.2) |
| Wood sandpiper | *Tringa glareola* | 2 (0.1) | - | - | - | 5 (0.3) | Y | 65 (6.4) |
| Marsh sandpiper | *Tringa stagnatilis* | Y | - | - | 20 (0.5) | 1 (0.1) | - | Y |
| Broad-billed sandpiper | *Limicola falcinellus* | Y | - | - | - | 2 (0.1) | - | - |
| Common sandpiper | *Actitis hypoleucos* | 2 (0.1) | - | - | - | 1 (0.1) | 35 (4.5) | 9 (0.9) |
| Terek sandpiper | *Xenus cinereus* | - | - | - | - | - | - | 1 (0.1) |
| Common redshank | *Tringa totanus* | Y | - | - | 66 (1.7) | 3 (0.2) | - | 14 (1.4) |
| Black-winged stilt | *Himantopus himantopus* | - | - | 10 (7.3) | 5 (0.1) | - | - | 22 (2.2) |
| Spotted redshank | *Tringa erythropus* | 4 (0.2) | - | - | - | 1 (0.1) | - | 15 (1.5) |
| Greenshank | *Tringa nebularia* | Y | - | - | - | - | Y | 1 (0.1) |
| Grey-tailed tattler | *Heteroscelus brevipes* | 2 (0.1) | - | - | - | - | - | - |
| Common snipe | *Gallinago gallinago* | - | - | - | - | - | - | 7 (0.7) |
| Snipe sp. | *Gallinago sp.* | Y | - | - | - | - | - | - |
| Pied avocet | *Recurvirostra avocetta* | 3 (0.2) | 10 (10.5) | 60 (43.8) | 62 (1.6) | 2 (0.1) | 4 (0.5) | 60 (5.9) |
| Grey plover | *Pluvialis squatarola* | 1 (0.1) | - | - | - | - | - | - |
| Little ringed plover | *Charadrius dubius* | Y | 4 (4.2) | - | 23 (0.6) | 25 (1.3) | Y | 15 (1.5) |
| Kentish plover | *Charadrius alexandrius* | - | - | 1 (0.7) | 38 (1) | - | - | 12 (1.2) |
| Oriental plover | *Charadrius veredus* | - | - | 2 (1.5) | 1 (0.0) | - | - | - |
| Northern lapwing | *Vanellus vanellus* | 200 (10.8) | Y | 2 (1.5) | 9 (0.2) | 4 (0.2) | - | 14 (1.4) |
| Eurasian hoopoe | *Upupa epops* | - | - | - | 10 (0.3) | 1 (0.1) | - | 1 (0.1) |
| Pacific swift | *Apus pacificus* | Y | - | - | Y | - | - | - |
| Common swift | *Apus apus* | - | - | - | - | - | 15 (1.9) | - |
| Isabelline shrike | *Lanius isabellinus* | - | - | - | Y | - | - | - |
| Eurasian crag martin | *Ptyonoprogne rupestris* | - | - | - | Y | - | - | - |
| Sand martin | *Riparia riparia* | Y | - | - | - | 1 (0.1) | - | - |
| Barn swallow | *Hirundo rustica* | - | - | - | Y | - | - | - |
| Horned lark | *Eremophila alpestris* | - | 1 (1.1) | 6 (4.4) | 1 (0.0) | 1 (0.1) | - | - |
| Mongolian lark | *Melanocorypha mongolica* | Y | 2 (2.1) | 2 (1.5) | - | 1 (0.1) | - | - |
| Eurasian Skylark | *Alauda arvensis* | - | - | - | Y | 1 (0.1) | - | - |
| Greater short-toed lark | *Calandrella cinerea* | - | - | - | - | - | - | Y |
| Lark sp. | *Calandrella/Alauda sp.* | Y | - | - | - | - | - | - |
| White wagtail | *Motacilla alba* | Y | - | - | - | - | - | 1 (0.1) |
| Yellow wagtail | *Motacilla flava* | - | - | - | Y | - | - | 7 (0.7) |
| Citrine wagtail | *Motacilla citreola* | - | - | - | Y | - | - | 3 (0.3) |
| Water pipit | *Anthus spinoletta* | - | 4 (4.2) | - | - | - | - | - |
| Isabelline wheatear | *Oenanthe isabellina* | - | Y | - | - | 2 (0.1) | Y | - |
| Northern wheatear | *Oenanthe oenanthe* | - | - | - | - | 1 (0.1) | Y | - |
| Rosy starling | *Sturnus roseus* | - | - | - | 1 (0.0) | - | - | - |
| White-cheeked starling | *Sturnus cineraceus* | - | - | - | - | - | - | 39 (3.8) |
| Common raven | *Corvus corax* | 2 (0.1) | Y | - | - | 1 (0.1) | 31 (4.0) | - |
| Black-billed magpie | *Pica pica* | - | Y | - | 10 (0.3) | - | - | 11 (1.1) |
| Daurian jackdaw | *Corvus dauuricus* | - | - | - | - | 1 (0.1) | - | - |
| Red-billed chough | *Pyrrhocorax pyrrhocorax* | - | Y | - | - | - | - | - |
| Tree sparrow | *Passer montanus* | Y | - | - | Y | - | - | - |
| House sparrow | *Passer domesticus* | - | - | - | Y | - | - | - |
|  | **Total** | **6,531**  **(351.9)** | **961**  **(1,011.6)** | **575**  **(419.7)** | **7,156 (187.3)** | **12,278 (661.5)** | **4,189**  **(537.1)** | **2,501**  **(246.9)** |
