# Supplementary material for: Highly Pathogenic Avian Influenza Virus among Wild Birds in Mongolia
Source: PLoS One. 2012 Sep 11;7(9):e44097. doi: 10.1371/journal.pone.0044097 (PMC3439473; doi:10.1371/journal.pone.0044097)
Supplement: Table S5 — Summary of information published relating to wild outbreaks of highly pathogenic avian influenza virus in Mongolia. (DOCX) [file pone.0044097.s005.docx]

**Online supporting information; Table S5.** Summary of information published relating to wild outbreaks of highly pathogenic avian influenza virus in Mongolia.

| **Start date** | **Location** | **Province** | **Latitude** | **Longitude** | **Number of deaths** | **Identity of birds reported** | **Sequences ^a^** | **Source** |
| --- | --- | --- | --- | --- | --- | --- | --- | --- |
| 31 July 2005 | Erhel Nuur | Khovsgol | 49.97 | 99.91 | 80 (+40) | 80 wild ducks, geese and swans reported through OIE. Also 16 dead birds estimated at less than a week old located during this study | A/WS/Mongolia/244/2005(H5N1)  A/WS/Mongolia/3/05(H5N1)  A/WS/Mongolia/4/05(H5N1)  A/WS/Mongolia/6/05(H5N1) | OIE WAHIS Ref: 5465, Sakoda pers. comm. 2011 |
| 31 July 2005 | Khunt Nuur | Bulgan | 48.43 | 102.58 | 9 | 9 wild ducks, geese and swans | A/BHG/Mongolia/1/05(H5N1) | OIE WAHIS Ref: 5465, Sakoda pers. comm. 2011 |
| 4 May 2006, 5 June 2006 | Khunt Nuur | Bulgan | 48.43 | 102.58 | 1 (+12) | 4 May 2006: A whooper swan found in a lake detected as part of the active surveillance in wild birds.  6 June 2006: 12 Migratory birds (gull, swan, goose). | A/WS/Mongolia/2/06(H5N1) | OIE WAHIS Ref: 4673 |
| 6 May 2006 | Erhel Nuur | Khovsgol | 49.97 | 99.91 | 1 | Common goldeneye | A/CG/Mongolia/12/2006(H5N1) | Unpublished data |
| 22 May 2009 | Doitiin Tsagaan Lake | Arkhangai | 47.34 | 102.32 | 9 (+1 destroyed) | A migratory swan | A/WS/Mongolia/1/2009(H5N1)  A/WS/Mongolia/2/2009(H5N1)  A/WS/Mongolia/4/2009(H5N1)  A/WS/Mongolia/5/2009(H5N1)  A/WS/Mongolia/6/2009(H5N1)  A/WS/Mongolia/7/2009(H5N1)  A/WS/Mongolia/8/2009(H5N1)  A/WS/Mongolia/9/2009(H5N1) | OIE WAHIS Ref: 8141 |
| 30 July 2009 | Erhel Nuur | Khovsgol | 49.97 | 99.91 | 3 (+1 sick) | Virus isolated from three dead juvenile Mongolian gulls and one sick Ruddy shelduck | CEIRS-UMN034-MN09-A-0833-T2.4 | Unpublished data |
| 29 July 2009 | Doroo Nuur | Arkhangai | 49.05 | 101.16 | 56 (1 Aug ’09)  171 (14 Aug ’09) | Anser indicus (bar-headed goose), Tadorna ferruginea (ruddy shelduck), Bucephala clangula (common goldeneye). | A/BHG/Mongolia/X25/2009(H5N1)  A/BHG/Mongolia/X53/2009(H5N1)  A/BHG/Mongolia/X54/2009(H5N1)  A/CG/Mongolia/X59/2009(H5N1)  A/CG/Mongolia/X60/2009(H5N1)  A/RSMongolia/X42/2009(H5N1)  A/RS/Mongolia/X63/2009(H5N1)  CEIRS-UMN034-MN09-A-0911-T2.4 | OIE WAHIS Ref: 8354, OIE WAHIS Ref: 8432  Sokoda pers. comm..2011 |
| 3 May 2010 | Ganga Nuur | Sukhbaatar | 45.27 | 113.99 | 26 | Whooper swans (Cygnus cygnus) and greylag geese (Anser anser) | A/WS/Mongolia/1/2010(H5N1)  A/WS/Mongolia/11/2010(H5N1)  A/WS/Mongolia/21/2010(H5N1) A/WS/Mongolia/7/2010(H5N1)  CEIRS-UMN034-MN10-A-0001-T2.4 | OIE WAHIS Ref: 9238 |

^a^ Abbreviated name of birds of each isolate: BHG: bar-headed goose, WS: whooper swan, CG: common goldeneye, RS: ruddy shelduck.
