# Supplementary material for: Highly Pathogenic Avian Influenza Virus among Wild Birds in Mongolia
Source: PLoS One. 2012 Sep 11;7(9):e44097. doi: 10.1371/journal.pone.0044097 (PMC3439473; doi:10.1371/journal.pone.0044097)
Supplement: Table S6 — Summary of information published relating to wild outbreaks of highly pathogenic avian influenza virus in China. (DOCX) [file pone.0044097.s006.docx]

**Online supporting information; Table S6.** Summary of information published relating to wild outbreaks of highly pathogenic avian influenza virus in China.

| **Start date** | **Location** | **Province** | **Latitude** | **Longitude** | **Number of deaths** | **Identity of birds reported** | **Source** |
| --- | --- | --- | --- | --- | --- | --- | --- |
| 4 May 2005  (30 April 2005)^b^ | Gangcha county, Quanji town, Niannaisuoma village | Qinghai |  |  | 519^a^  1,500^b^  6,184^c^ | Migratory birds, including bar-headed geese (*Anser indicus*), great black-headed gulls (*Larus ichthyaetus*), brown-headed gulls (*Larus brunnicephalus*), ruddy shelducks (*Tadorna ferruginea*) and great cormorants (*Phalacrocorax carbo*) | ^a^OIE (ISSN 1012-5329)  ^b^[1]  ^c^[2] |
| 26 May 2006 | Dangxiong | Tibet | 31.7338 | 87.2254 | 57 | Migratory birds | OIE WAHIS Ref: 4904 |
| 26 May 2006 | Lhasa | Tibet | 31.6319 | 88.3465 | 28 | Migratory birds | OIE WAHIS Ref: 4904 |
| 21 May 2006 | Naqu (district) | Tibet | 31.9376 | 89.2637 | 2,579 | 300 bar-headed geese, 65 widgeons, 6 crows, 6 hawks, 1 black-necked crane and 82 other wild birds | OIE WAHIS Ref: 4904 |
| 21 May 2006 | Guolou (district) | Qinghai | 35.3008 | 96.4995 | 451 | 235 bar-headed geese, 5 brown-headed gulls, 23 ruddy shelducks, 9 grebes, 3 owls, 1 black-neck crane, 6 common cormorants, 1 grassland vulture and 2 condors | OIE WAHIS Ref: 4904 |
| 23 April 2006 | Yushu (county) | Qinghai | 35.6065 | 95.8881 | 533 | 404 bar-headed geese, 3 brown-headed gulls, 4 ruddy shelducks, 1 tern, 6 grebes, 1 egret, 2 goosanders, 2 *Casmerodius* | OIE WAHIS Ref: 4904 |
| 8 May 2009 | Genggahu Lake | Qinghai | 36.6 | 99.16 | 121 | Dead wild birds | OIE WAHIS Ref: 8107 |
| 27 May 2009 | Nanhai Prefecture | Qinghai | 36.6 | 99.1 | 162 | Dead wild birds | OIE WAHIS Ref: 8155 |
| 9 May 2010 | Shuanghu district | Tibet | 32.422 | 89.206 | 170 | 141 brown-headed gulls (*Larus brunnicephalus*), 27 bar-headed geese (*Anser indicus*), one red-billed chough (*Pyrrhocorax pyrrhocorax*) and one Eurasian wigeon (*Anas penelope*). | OIE WAHIS Ref: 9470 |

1. Chen H, Smith GJD, Zhang SY, Qin K, Wang J, et al. (2005) H5N1 virus outbreak in migratory waterfowl. Nature 436: 191–192.

2. Chen HL, Li YB, Li ZJ, Shi JZ, Shinya K, et al. (2006) Properties and dissemination of H5N1 viruses isolated during an influenza outbreak in migratory waterfowl in western China. J Virol 80: 5976–5983.
