# Supplementary material for: Highly Pathogenic Avian Influenza Virus among Wild Birds in Mongolia
Source: PLoS One. 2012 Sep 11;7(9):e44097. doi: 10.1371/journal.pone.0044097 (PMC3439473; doi:10.1371/journal.pone.0044097)
Supplement: Table S7 — Summary of information published relating to wild outbreaks of highly pathogenic avian influenza virus in the Tyva Republic, Russian Federation. (DOCX) [file pone.0044097.s007.docx]

**Online supporting information; Table S7.** Summary of information published relating to wild outbreaks of highly pathogenic avian influenza virus in the Tyva Republic, Russian Federation.

| **Start date** | **Location** | **Province** | **Latitude** | **Longitude** | **Number of deaths** | **Identity of birds reported** | **Source** |
| --- | --- | --- | --- | --- | --- | --- | --- |
| 10 Jun 2006 | Uuvs Nuur | Tyva | 50.6 | 93.0 | 3,262 | Common pochard (*Aythya ferina*) and great crested grebe (*Podiceps cristatus*) were most abundant dead birds. Great cormorant (*Phalacrocorax carbo*), common coot (*Fulica atra*), common tern (*Sterna hirundo*) and black-headed gull (*Chroicocephalus ridibundus*) died in smaller numbers from 20 June onward. | [1] |
| 11 June 2009 | Uuvs Nuur | Tyva | 50.6 | 93.0 | 58^a^ | 10 dead birds belonging to 4 species (great crested grebe [*Podiceps cristatus*], little grebe [*Tachybaptus ruﬁ collis*], black-headed gull [*Larus ridibundus*], and spoonbill [*Platalea leucorodia*])^b^ | ^a^OIE WAHIS Ref: 8220  ^b^[2] |
| 5 June 2010 | Uuvs Nuur | Tyva | 50.6 | 93.0 | 367 | Wild birds found dead on a lake | OIE: 9451 |

1. Lvov DK, Shchelkanov MY, Deryabin PG, Fedyakina IT, Burtceva EI, et al. (2006) Isolation of highly pathogenic avian influenza (HPAI) A/H5N1 strains from wild birds in the epizootic outbreak on the Uvs-Nur Lake (June 2006) and their incorporation to the Russian Federation state collection of viruses. Vopr Virusol 51: 14–18. [In Russian]

2. Sharshov K, Silko N, Sousloparov I, Zaykovskaya A, Shestopalov A, et al. (2010) Avian Influenza (H5N1) Outbreak among Wild Birds, Russia, 2009. Emerging Infectious Diseases 16: 349–351. doi:10.3201/eid1602.090974.
